# SARS-CoV-2 detection using a nanobody-functionalized voltammetric device

**DOI:** 10.1038/s43856-022-00113-8

**Published:** 2022-05-23

**Authors:** Quentin Pagneux, Alain Roussel, Hiba Saada, Christian Cambillau, Béatrice Amigues, Vincent Delauzun, Ilka Engelmann, Enagnon Kazali Alidjinou, Judith Ogiez, Anne Sophie Rolland, Emmanuel Faure, Julien Poissy, Alain Duhamel, Rabah Boukherroub, David Devos, Sabine Szunerits

**Affiliations:** 1 Univ. Lille, CNRS, Centrale Lille, Univ. Polytechnique Hauts-de-France, UMR 8520 - IEMN, Lille, France; 2 Laboratoire d’Ingénierie des Systèmes Macromoléculaires (LISM), Institut de Microbiologie, Bioénergies et Biotechnologie (IM2B), Aix-Marseille Université - CNRS, UMR, Marseille, France; 3 Univ Lille, CHU Lille, Laboratoire de Virologie ULR3610, Lille, France; 4 Univ. Lille, CHU-Lille, Inserm, U1172, Lille Neuroscience & Cognition, LICEND, Lille, France; 5 Service Universitaire de maladies infectieuses - Hôpital Hutiez, CHU de Lille, Lille, France; 6 UMR8204 U1019, Centre infection et immunité de Lille, Equipe Opinfield, Institut Pasteur de Lille, Lille, France; 7 Univ. Lille, Inserm U1285, CHU Lille, Pôle de réanimation, CNRS, UMR 8576 - UGSF - Unité de Glycobiologie Structurale et Fonctionnelle, Lille, France; 8 Univ. Lille, CHU Lille, ULR2694 METRICS: évaluation des technologies de santé et des pratiques médicales, Lille, France

**Keywords:** Nanobiotechnology, Diagnostic markers

## Abstract

**Background:**

An ongoing need during the COVID-19 pandemic has been the requirement for accurate and efficient point-of-care testing platforms to distinguish infected from non-infected people, and to differentiate SARS-CoV-2 infections from other viruses. Electrochemical platforms can detect the virus via its envelope spike protein by recording changes in voltammetric signals between samples. However, this remains challenging due to the limited sensitivity of these sensing platforms.

**Methods:**

Here, we report on a nanobody-functionalized electrochemical platform for the rapid detection of whole SARS-CoV-2 viral particles in complex media such as saliva and nasopharyngeal swab samples. The sensor relies on the functionalization of gold electrode surface with highly-oriented Llama nanobodies specific to the spike protein receptor binding domain (RBD). The device provides results in 10 min of exposure to 200 µL of unprocessed samples with high specificity to SARS-CoV-2 viral particles in human saliva and nasopharyngeal swab samples.

**Results:**

The developed sensor could discriminate between different human coronavirus strains and other respiratory viruses, with 90% positive and 90% negative percentage agreement on 80 clinical samples, as compared to RT-qPCR.

**Conclusions:**

We believe this diagnostic concept, also validated for RBD mutants and successfully tested on Delta variant samples, to be a powerful tool to detect patients’ infection status, easily extendable to other viruses and capable of overcoming sensing-related mutation effects.

## Plain language summary

Our ability to control SARS-CoV-2, the virus that causes COVID-19, relies on access to rapid and sensitive technologies that can detect the virus, and differentiate SARS-CoV-2 infections from infections with other viruses. One potential approach to detect SARS-CoV-2 is to use sensors comprising specific surfaces and chemicals that can detect changes in the properties of samples containing the virus. Here, we develop one of these sensors and test it in samples from people with COVID-19 or other viral infections, or people without. We show that our portable device can quickly and accurately detect SARS-CoV-2 and can also detect different variants. We also show that signals from our device are specific for SARS-CoV-2. Our device may provide another tool for COVID-19 diagnosis, with benefits being portability and speed compared with current gold-standard approaches.

## Abstract (web summary)

Pagneux et al. developed a nanobody-functionalized electrochemical platform with smartphone readout to detect SARS-CoV-2 in clinical samples. They demonstrated that their device can rapidly and accurately detect SARS-CoV-2 in saliva and nasopharyngeal swab samples and discriminate between this virus and other respiratory viruses.

## Introduction

Diagnosis of the highly contagious Coronavirus disease 2019 (COVID-19), caused by severe acute respiratory syndrome-coronavirus-2 (SARS-CoV-2), remains largely based on reverse transcription PCR (RT-qPCR), which identifies the genetic material of the virus in the nasopharyngeal area or in saliva^1,2^. The advantage of RT-qPCR is high sensitivity^3,4^ with limitations being assay time and cost-related issues^4,5^. Rapid electrochemical detection of SARS-CoV-2 based on isothermal rolling cycle amplification with probes that were functionalized with redox active labels was recently proposed by Chaibun et al. allowing in less than 2 h to detect 1 copy µL^−1^ of N and S genes^6^. Time-related issues can be further overcome by rapid antigenic tests where the presence of SARS-CoV-2 is detected using surface anchored antibodies that recognize and attach to the viral spike antigen^7–13^. Although faster and cheaper compared to RT-PCR assays, rapid antigenic tests are currently less sensitive^9,14^. The several positive aspects of antigenic tests being simple to implement, prompted us to look for an electrochemical alternative of high sensitivity, which can easily be mass-produced and implemented in clinical settings and as point of care devices. Indeed, electrical and electrochemical platforms own qualitative and quantitative sensing capacity all together in a user-friendly sensing format^8–11,15–19^. An original detection of COVID-19 using an organic electrochemical transistor was recently reported by the group of Inal and co-workers^10^. The combination of a solution-processable conjugated polymer as a transistor channel together with nanobody-SpyCatcher fusion protein surface receptors allows SARS-CoV-2 spike protein detection in nasopharyngeal swab samples of different viral loads. Nevertheless, electronic-based protein sensors translate still poorly into market products due to complex sensor design.

We report here a highly performing electrochemical COVID-19 detection approach with following key features: (1) the use of an engineered SARS-CoV-2 specific nanobody as surface receptor, (2) controlled non-fouling surface biofunctionalization, (3) the ability to sense the infectivity of a patient by using the spike protein (S1) as target, and (4) quantification via differentical pulse voltammetry (DPV) read out. We found VHH-72-13C to be most adapted surface receptor for the electrochemical “signal off” diagnostic assay using ferrocenemethanol as a redox mediator. Integration of VHH-72-13C onto a poly(ethylene)glycol-modified gold electrode via maleimide-thiol linkage chemistry reliably and specifically detects SARS-CoV-2 viral particles electrochemically with a limit of detection of LoD = 1.2 × 10^4^ viral RNA copies mL^−^^1^, corresponding to a Ct value of 33 using cultured SARS-CoV-2 virus particles and correlates to about 2 ± 1 PFU mL^−1^. The sensing technology remains operational on recognizing RBD mutations including clades related to the Alpha, Beta and Delta variants.

The performance of the sensor was validated in a study on 80 patients (40 positive, 40 negative) unprocessed nasopharyngeal and saliva samples. The in vitro diagnostic device showed a 90% positive percentage agreement (PPA) and a 90% negative percentage agreement (NPA), as compared to RT-qPCR for nasopharyngeal samples. In the case of saliva, a 80% PPA and a 85% NPA were determined when compared to RT-qPCR for nasopharyngeal samples of the same patients. The portability of the sensor and its read out, which can be directly connected to a mobile telephone completes the electrical signal processing, making it user-friendly and operational in different situations and environments. This technology is broadly applicable and only limited by the availability of nanobodies targeting the antigen of interest.

## Methods

### Materials

3-mercaptopropionic acid (98%, Ref: M5801), 1-ethyl-3-[3-dimethylaminopropyl]-carbodiimide hydrochloride) (EDC, Ref: E7750), N-hydroxysuccinimide (NHS, Ref. 130672), and ferrocenemethanol (97%, Ref. 335061) were purchased from Sigma Aldrich and used as-received. Phosphate saline solution (PBS, 1×, Ref. 10010-015) was obtained from Thermo Fisher scientific. Maleimide-PEG_x_-amine (MW 1 kDa, Ref. LV3811) was purchased from Interchim Uptima. MQ-water was used throughout the whole study.

Universal transport Medium (UTM) was obtained from Copan, Italy (UMT-RT). The formulation of UTM-RT medium includes protein for stabilization, antibiotics to minimize bacterial and fungal contamination, and a buffer to maintain a neutral pH.

Screen printed electrodes were obtained from IPM -Intelligent Pollutant Monitoring Denmark, AUH3600, via Hdts France and consist of a 4 mm diameter gold electrode, and a silver/silver chloride reference and carbon counter electrode. Disposable PD 10 Desalting Columns (ref. 17–0851-01.) were purchased from Cytiva, France.

### Surface modification approaches

#### Direct immobilization of VHH-72-C13

The as-received Au electrodes were exposed to 20 µL of VHH-72-C13 (0.1 mg/mL) for 12 h at 4 °C. The surface was washed with MQ-water and dried with an air duster. The resulting modified surface was washed copiously with MQ-water to remove excess nanobody.

#### Immobilization of VHH-72-C13 and VHH-H11D4-13C via PEG units

The Au electrodes were exposed to 10 µL of an aqueous solution of 3-mercaptopropionic acid (25 mM) for 30 min at room temperature. The surface was washed with MQ-water and dried with an air duster. Then, the acid-terminated surface was activated with EDC/NHS (1:1 molar ratio, 15 mM) for 20 min, followed by immersion into NH_2_-PEG_6_-maleimide (10 µL, 0.1 mg/m, in PBS 1×) for 2 h at 4 °C and washing with MQ-water. The interface was then modified with the VHH-72-C13 or VHH-H11D4-13C nanobody (10 µL, 0.1 mg/mL, PBS 1×)) for another 2 h at 4 °C. The resulting modified surface was washed copiously with MQ-water to remove excess nanobodies and unreacted reagents, and then stored at 4 °C before use.

#### Immobilization of VHH-72-C13 via 3-mercaptoproponic acid

The as-received gold electrodes were functionalized with 20 µL of an aqueous solution of 3-mercaptopropionic acid (25 mM) for 30 min at room temperature. The surface was washed with MQ-water and dried with an air duster. The interface was treated with EDC/NHS (1:1 molar ratio, 15 mM) for 20 min, followed by the addition of VHH-72 (20 µL, 0.1 mg mL^−1^) for 2 h at room temperature. The VHH-72 modified electrode was washed with water, and stored at 4 °C for further use.

### Surface modification with 6-(ferrocenyl) hexanethiol

Attachment of 6-(ferrocenyl) hexanethiol to Au-PEG_6_-MAL electrodes: Au-PEG_6_-MAL interfaces were coated with 200 µL of 6-(ferrocenyl) hexanethiol (100 µg mL^−1^) for 2 h followed by washing (three times) with ethanol and MQ water (three times).

### Characterisation

**X-ray photoelectron spectra (XPS)** were recorded with an SPECSLAB II (Phoibos-Hsa 3500 150, 9 channels) SPECS spectrometer with Al Kα source (E = 1486.6 eV) operating at 12 kV, pass energy (E_pass_ = 40 eV), 0.1 eV energy step and acquisition time of 1 s per point. The residual pressure inside the analysis chamber was ∼1 × 10^−8^ Torr. All XPS were referenced according to the adventitious C1s peak at 284.5 eV.

**Electrochemical measurements** were performed with a Sensite Smart smartphone potentiostat (Palmsense, The Netherlands, distributed by HDts in France). Differential pulse voltammograms (DPV) were recorded at the appropriate potential range using following DPV parameters: t_aqu_ = 3 s, E_step_ = 0.01 V, E_pulse_ = 0.06 V, t_pulse_ = 0.02 V, scan rate = 0.06 V s^−1^. The active surface area of naked gold was determined to be 0.127 cm^2^.

### Working principle of the sensor

The sensors were incubated for 10 min in 200 µL of sample in the collection vial and the solution was agitated by hand gently to increase mass transport. Longer times did not result in further change of the electrochemical signal thereafter. The incubation step was followed by washing in PBS (1×). To this interface, 200 µL of fresh ferrocenemethanol (1 mM, PBS 1×) was added and a DPV was recorded. The difference of the maximal current before and after contact with the sample was used to discriminate between positive and negative samples. A current density difference of 2 µA was used as a cut-off value to differentiate between both cases.

In the case of analysis of saliva samples, the electrochemical measurements were performed immediately (5–15 min) after sample collection to minimize protease degradation of the samples.

### Nanobody production

#### The full lenght sequence of VHH72-C13 is as follows

MKYLLPTAAAGLLLLAAQPAQVQLQESGGGLVCAGGSLRLSCAASGRTFSEYAMGWFRQAPGKEREFVATISWSGGSTYYTDSVKGRFTISRDNAKNTVYLQMNSLKPDDTAVYYCAAAGLGTVVSEWDYDYDYWGQGTQVTVSSGSHHHHHH

The N-terminal PelB leader sequence is highlighted in grey, the cysteine mutation is in red and the C-terminal purification tag is bold.

VHH-72-C13 was ordered as synthetic codon-optimized genes in pET24 vector by Twist Biosciences for production in *E. coli*. Genes were fused with the N-terminal PelB leader sequence (MKYLLPTAAAGLLLLAAQPA) for periplasmic protein expression and with a C-terminal purification His-tag. pET24 plasmid contains an inducible T7 promoter with isopropyl β-d-1-thiogalactopyranoside (IPTG) and kanamycin resistance.

VHH-72-C13 was also synthetized as a gBlocks codon-optimized gene fragment by IDT Technologies for production with HEK293 mammalian cells and fused with a C-terminal purification His-tag. Gene fragment was introduced into pYD11 expression vector by using the In-Fusion HD cloning kit (Takara). pYD11 plasmid contains a CMV promotor and ampicillin resistance, an IgK signal peptide (METDTLLLWVLLLWVPGSTG) for VHH secretion and a human Fc-tag downstream the cloning site. All cloned sequences were verified by DNA sequencing from Eurofin Genomics. Plasmids were then amplified under antibiotic selection after transformation of *E. coli* RapidTrans TAM1 competent cells (Active Motif). Plasmids were purified by using a NucleoBond Xtra Maxi plus kit (Macherey-Nagel). The plasmid is not available via Addgene, but is available from the authors upon request.

VHH-72-C13 and VHH-H11D4-C13 were produced in Rosetta (DE3) pLysS *E. coli* cells (Novagen) cultured in Turbo Broth media (AthenaES) at 37 °C up to an OD_600_ of 0.6. Cells were then induced with 0.1 mM IPTG. At this stage, the temperature was decreased to 28 °C and cells grew for an additional 18 h. Cells were harvested by centrifugation (4000 g for 10 min) and the pellet was homogenized and frozen in lysis buffer (50 mM Tris pH 8.0, 300 mM NaCl, 10 mM imidazole, 0.1 mg/mL lysozyme, 1 mM phenylmethylsulfonyl fluoride). After thawing, DNAse I (20 μg/mL) and MgSO_4_ (1 mM) were added and cells were lysed by sonication. The pellet and soluble fractions were separated by centrifugation (16,000 g for 30 min). VHH-72-C13 were purified from the soluble fraction on immobilized metal ion affinity chromatography using a 5 mL HisTrap crude Ni^2+^-chelating column (GE Healthcare) equilibrated in buffer A (50 mM Tris pH 8.0, 300 mM NaCl, 10 mM imidazole). VHH was eluted with buffer A supplemented with 250 mM imidazole and was further purified by a size exclusion chromatography (HiLoad 16/60 Superdex 75 prep grade, GE Healthcare) equilibrated in PBS buffer. Purity of the protein was monitored at all stages of the purification process using SDS-PAGE and visualized by Coomassie blue staining.

### Biological experiments

#### Vero E6 cells

(ATCC CRL-1586) were cultured in Dulbecco’s modified Eagle medium (DMEM) supplemented with 10% fetal bovine serum (FBS), 1% L-glutamine, 1% antibiotics (100 U mL^−1^ penicillin, and 100 μg mL^−1^ streptomycin), in a humidified atmosphere of 5% CO_2_ at 37 °C.

#### Virus titration

Vero E6 cells were plated in 96-well plates (2.5 × 10^5^ cells/well) 24 h before performing the virus titration. A clinical isolate, obtained from a SARS-CoV-2 positive specimen, was cultured on Vero E6 cells. Infected cell culture supernatant was centrifuged for 10 min at 1500 rpm at 4 °C to obtain a virus suspension. The virus suspension was used undiluted and in serial ten-fold dilutions (10^−1^ to 10^−9^). Virus suspensions were distributed in 6 wells in DMEM supplemented with 10% FBS (Fetal Bovine Serum) to Vero E6 cells, 1% antibiotics (100 U mL^−1^ penicillin, and 100 μg mL^−1^ streptomycin), and 1% L-glutamine. The plates were incubated for 6 days in 5% CO_2_ atmosphere at 37 °C. The plates were examined daily using an inverted microscope (ZEISS Primovert) to evaluate the extent of the virus-induced cytopathic effect in cell culture. Calculation of estimated virus concentration was carried out by the Spearman and Karber^20,21^ method and expressed as TCID_50_/mL (50% tissue culture infectious dose). TCID_50_/mL values were transformed to PFU/mL by using the formula PFU/mL = TCID_50_/mL × 0.7.

#### SARS-CoV-2 RT-PCR

A real-time RT-aPCR method developed by the French Reference Center for respiratory viruses (Institut Pasteur, Paris)^22^ was used. This method is a duplex RT-PCR targeting two regions in the RdRp gene, namely IP2 and IP4. G6PDH RT PCR using primers G6PDH-6(GAAGGTGAAGGTCGGAGT), G6PDH-231(GAAGATGGTGATGGGATTTC) and the probe G6PDH-202(5′FAM-CAAGCTTCCCGTTCTCAGCC-3′BHQ) was additionally performed to monitor for specimen quality, extraction and PCR inhibition. Undetectable SARS-CoV-2 levels were set to Ct 40. Amplification was performed on 7500 Real-Time PCR System (Applied Biosystems, USA).

#### PCR of other viruses

Briefly, nucleic acid isolation from respiratory specimens was carried out with the STARMag Universal Cartridge Kit on the Microlab NIMBUS instrument (Seegene, Seoul, South Korea), and virus detection was done using the Allplex™ Respiratory assay (Panels 1, 2, and 3) (Seegene) on a CFX96 thermal cycler (Biorad, Marnes-la-Coquette, France).

### Clinical study

Cor-Dial-1 study “Rapid Detection of COVID-19 by Portable and Connected Biosensor: Biological Proof of Concept” is a case control study that prospectively enrolled 200 participants from consultation (outpatient), hospitalisation and intensive care unit (mean age: 46 ± 22 years (min = 1 year: max = 95 years), sex ratio male/female:1.07) including the 100 first people with a positive diagnosis of COVID-19 and the 100 first people with a negative diagnosis of COVID-19 defined by RT-qPCR by the medical team (from August to December 2020). The same nasopharyngeal swabs were used for COVID-19 RT-PCR and for the viral sensor. The final diagnosis of COVID-19 was planned to be independently performed by the medical team (EF and JP) using the notion of infectious contagion, the clinical signs, the pulmonary CT scan and the RT-qPCR of SARS-CoV-2 and sometimes the serology and a second RT-qPCR test for negative cases suspected to be positive. However, in this series, all the RT-qPCR positive cases were considered COVID-19 and all the negative RT-qPCR were considered not to be COVID-19. Consequently, only percentage of concordance between this study and RT-qCR were calculated (no Cohen’s Kappa Coefficient for concordance) and the sensitivity and specificity of the sensor.

Our study (Ref Protocol: 2021/0063; Ref IDRCB 2021-A00387-34; Ref promotor: CHU of Lille: 21.02.11.57302) was approved by the independent ethics committee of Iles de France Paris IX on 7th of April 2021 (REF No. 2021/22) as a type 3. The study has been registered on ClinicalTrials.gov ID: NCT04780334. All patients provided written, informed consent before inclusion.

### Statistics and reproducibility

Three technical replicates were taken on the same sample to validate the reproducibility of the approach. The mean (±SEM) was traced in all the experiments.

### Reporting summary

Further information on research design is available in the Nature Research Reporting Summary linked to this article.

## Results and discussion

### Biofunctionalization of gold electrodes with nanobodies

**Fig. 1:**
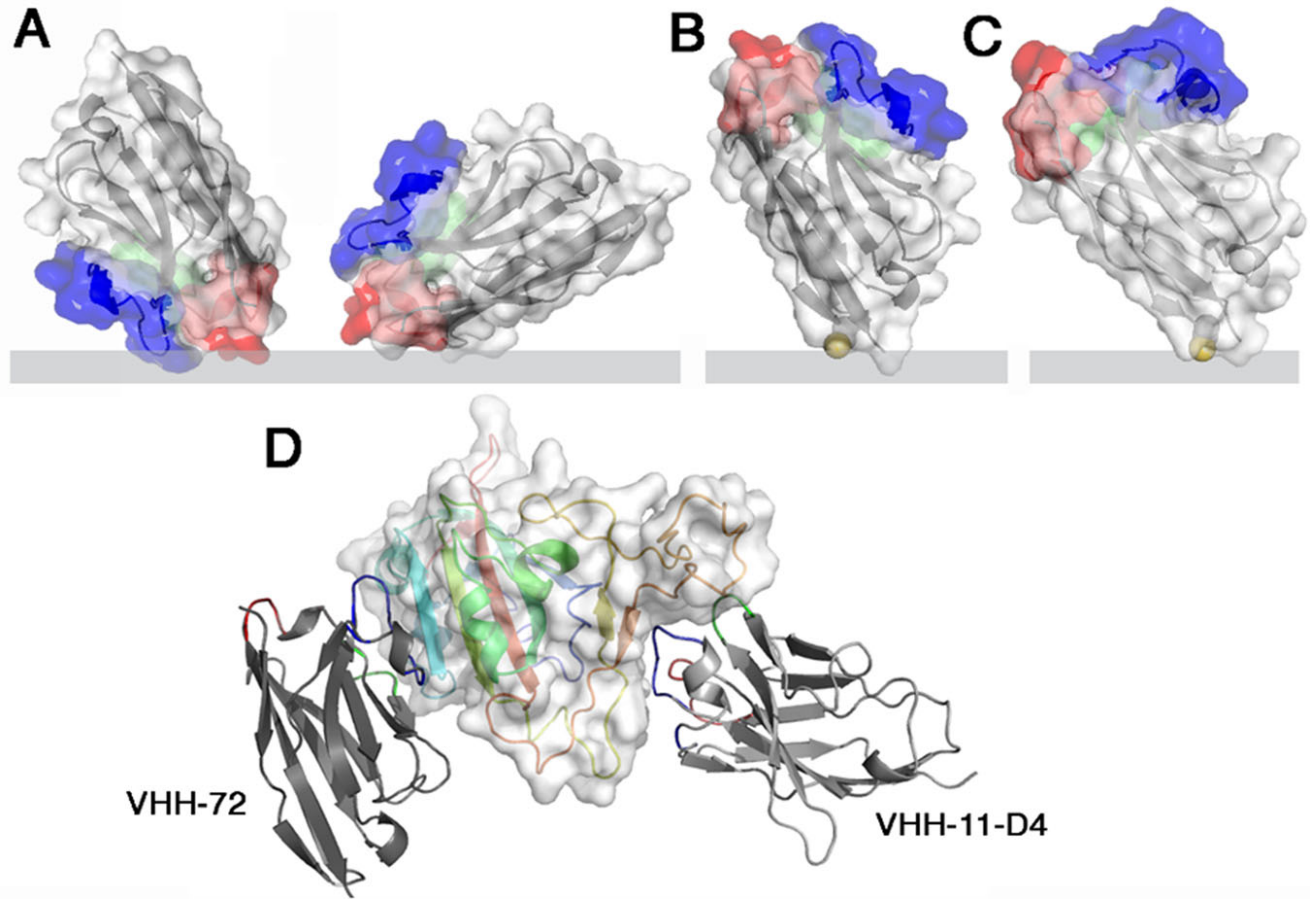
Different generations of nanobodies were used in this work and integrated into the electrochemical sensing device. Surface and ribbon views of the VHHs (the three CDRs are colored red, green, blue, respectively): **A** VHH-72 can bind the sensor in different orientations. **B** VHH-72-C13 is oriented on the sensor by the Cys link (yellow). The direct linkage to the gold electrode is shown rather than the thiol-PEG-MAL linker, considered more meaningful. **C** VHH11-D4-C13 is oriented on the sensor surface by the Cys link (yellow). **D** Ribbon views of the two VHHs bound to the Spike RBD (rainbow colored, white surface). Note the large distance between the two epitopes. VHHs affinity constants determined by BLI: VHH-72 (K_D_ = 36.6 nM), VHH-72-13C (K_D_ = 12.1 nM), VHH-H11D4-13C (K_D_ = 5 nM).

A common drawback of biosensors relates to the immobilization of the receptor onto the transducer surface in a controlled manner without loss of its recognition property. Immunoglobulin or Fab fragments are favourite binder candidates. However, using a full-length immunoglobulin or even a Fab fragment could be detrimental to an optimum electrochemical detection, as the distance from the binder’s attachment point to the antigen binding site is large and might result in diffusion limited access of redox mediators to the surface. To overcome this hurdle, we opted here for the use of engineered nanobodies (Fig. 1), which correspond to antibodies devoid of light chain and lacking CH1 domain^23,24^. This variable domain alone, with no associated light domain, performs the recognition function and possesses several key features such as small size (i.e., 4 nm in length, 2.5 nm in width, and about 15 kDa in molecular weight), high solubility, high chemical stability, and ease of production that attracted their increasing interest for sensing^25,26^. The improved shelf-life of nanobodies over antibodies with retained antigen-binding capacity makes them, in addition, ideal building blocks for sensing devices as the one described here^27^.

We selected VHH-72 (PDB ID 6WAQ), an anti SARS-CoV-1 anti-spike nanobody^28^, which cross-neutralizes SARS-CoV-2, for our sensing strategy. However, while stable nanobody-gold conjugates can be generated via physical adsorption of VHH-72 based on electrostatic interactions^29^, careful consideration of the influence of the isoelectric point, and ionic strength of the solution limits biosensing in complex media such as nasopharyngeal swab samples and saliva. Random attachment of VHH-72 is also most likely to decrease the binding efficiency of a bulky target such as the SARS-CoV-2 viral particle. Orientation of the VHH-72 nanobody, in a position where it would be roughly perpendicular to the chip’s surface, is much more desirable and can be achieved by engineering VHH-72 through introduction of a cysteine mutant in a loop between the frameworks 1 and 2, opposite to the binding site, at position 13 (Fig. 1). VHH-72-C13 was expressed in *Escherichia coli’s* periplasm and purified in two steps by affinity and size exclusion chromatography (Supplementary Fig. S1). No cysteine cross-linking was observed (Supplementary Fig. S1). Wrapp and co-authors reported that VHH-72 displayed high affinity (K_D_ = 36.6 nM) towards SARS-CoV-2 S protein^28^. Our SPR measurements (Supplementary Fig. S2) with VHH-72-C13 and SARS-CoV-2 RBD revealed a K_D_ of 12.1 nM.

**Fig. 2:**
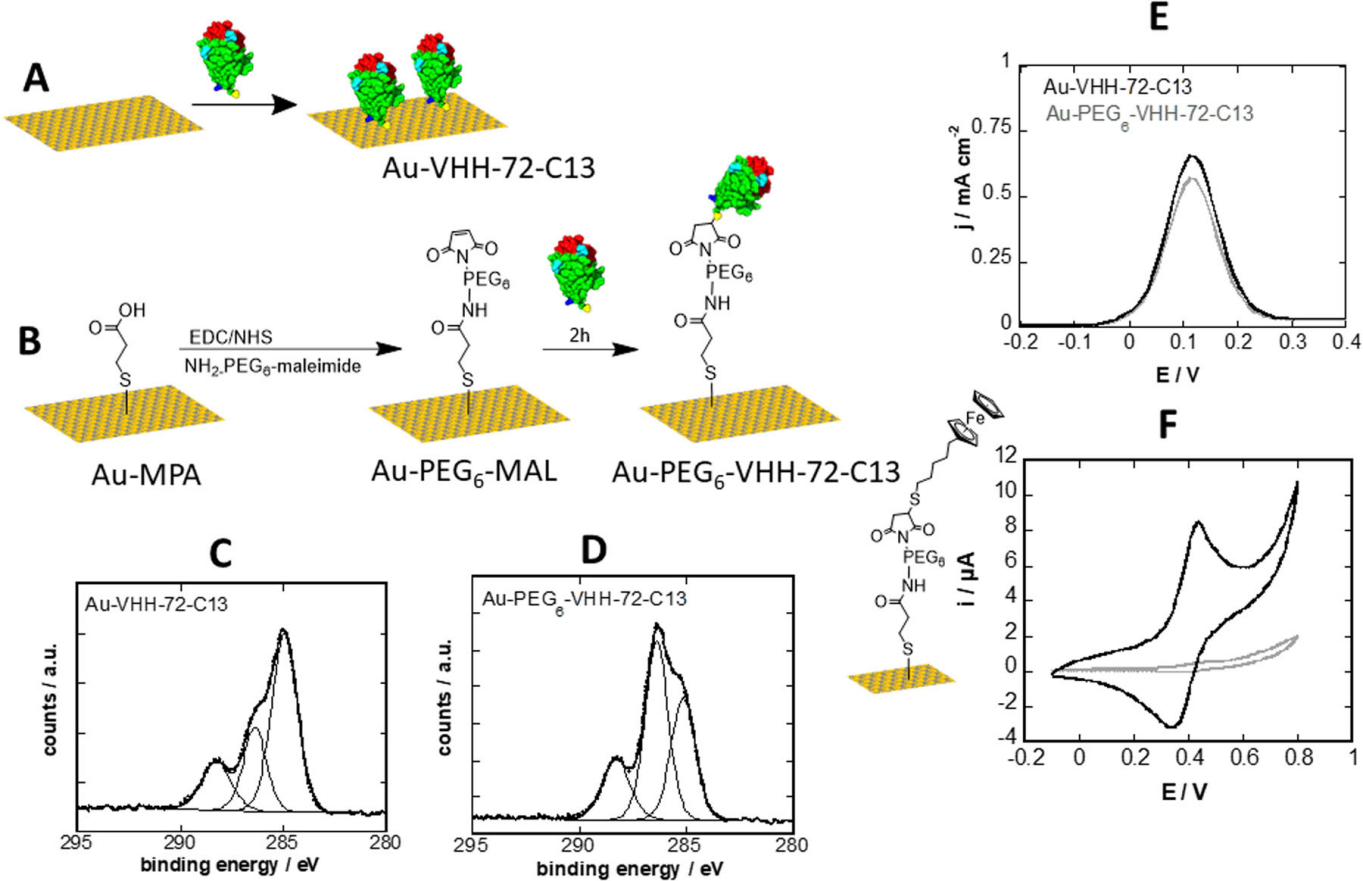
Design and characterisation of VHH-72-13C modified gold electrodes. **A** Direct linkage via cysteine groups of VHH-72-13C. **B** Maleimide-thiol based grafting onto a functional PEG_6_ spacer. **C** C_1s_ high resolution XPS spectra of Au-VHH-72-C13 and **D** Au-PEG_6_-VHH-72-C13 surfaces. **E** Differential pulse voltammograms (DPVs) of Au-VHH-72-C13 (black) and Au-PEG_6_-VHH-72-C13 (grey) in ferrocenemethanol (1 mM in 0.1 M PBS, pH 7.4). **F** Au-PEG_6_-MAL interface modified with 6-(ferrocenyl) hexanethiol and the corresponding cyclic voltammogram in PBS (0.1 M PBS, pH 7.4). Control: immobilization of ferrocene (grey line) scan rate: 100 mV s^−1^.

VHH-72-13C monolayers were formed by immersing freshly cleaned gold electrodes into a solution of VHH-72-13C in PBS for 24 h, providing the required time for the formation of gold-thiolate bonds between the gold substrate and the cysteine mutant (Fig. 2A).

We further opted for immobilization of VHH-72-13C via a 3-mercaptopropionic acid (MPA) self-assembled monolayer post-functionalized with a bifunctional maleimide modified poly(ethylene glycol) (PEG_6_) spacer ligand (Fig. 2B). This construct was incubated under physiological conditions with VHH-72-13C for 2 h, resulting in covalent linkage of the nanobody on the surface. The presence of the PEG_6_ spacer is favourable to overcome eventual limitation in virus binding to VHH-72-13C due to steric hindrance and its hydrophilic character limits biofouling from non-specific adsorption of proteins and cells^24,30^. The immobilization steps (MPA, PEG_6_-MAL, VHH) were monitored through X-ray photoelectron spectroscopy (XPS) (Fig. 2C, D) and differential pulse voltammogram (DPV) measurements (Fig. 2E). Upon functionalization with VHH-72-13C, band of C_1s_, O_1s_, N_1s_, S_2p_ and Au_4f_ (Table S1) were observed. The high resolution C_1s_ XPS spectrum of Au-VHH-72-C13 reveals bands at 285.0 eV (C–C/C–H), 286.7 eV (C–S/C–O/C–N) and 288.6 eV (O–C=O/N–C=O) (Fig. 2C). The C_1s_ of the Au-MPA surface displays bands at 285.0 eV (C–C/C–H), 286.7 eV (C–S/C–O) and 288.2 (O-C=O) eV (Supplementary Fig. S3) with Au-PEG_6_-MAL showing similar deconvolution at 285.0 eV (C–C/C–H), 286.3 eV (C–O) and 288.7 eV (O–C=O and N–C=O) (Supplementary Fig.Supplementary Fig. S3). The Au-PEG_6_-VHH-72-C13 interface (Fig. 2D) indicates a significant change in the C_1s_ feature with bands at 285.0 eV (C–C/C–H), 286.3 eV (C–O, C–N, C–S) and 288.2 eV (O–C=O and N–C=O). The increase of the band at 286.3 eV is due to the introduction of additional C–N and C–S bonds brought by the VHH-72-C13. In all cases, signals of the underlying Au_4f_ are visible, indicating that surface modifications result in film thicknesses below 10 nm and/or that the gold electrode is not fully covered.

Differential pulse voltammograms (DPVs) recorded on Au-VHH-72-C13 and Au-PEG_6_-VHH-72-C13 electrodes using ferrocenemethanol as a redox mediator display well-defined redox peaks with a maximum current density at E = + 0.12 V vs. Ag/AgCl (Fig. 2E). To determine the packing density of VHH-72-C13 on the PEGylated surface, 6-(ferrocenyl) heaxanethiol (100 µg mL^−1^) was integrated onto Au-PEG_6_-MAL. From the cyclic voltammogram (CV) (Fig. 2F), a surface coverage of Γ = (2.7 ± 1.2) × 10^−11^ mol cm^−2^ was determined using the relation Γ=Q/nFA where Q is the passed charge (C), n the number of exchanged electrons (*n* = 1), F the Faraday constant (96485 C mol^−1^) and A the electroactive surface of the electrode. This correlates to (16.3 ± 3.2) × 10^12^ molecules coupled per cm^2^. Considering the ferrocene molecules as spheres with a diameter of 6.6 Å, the theoretical maximum coverage for an idealized ferrocene monolayer is estimated as Γ = 4.4 × 10^−10^ mol cm^−2^ ^31^. Correlating it to the size of nanobody domain (4 × 2.5 nm), a maximal coupling density physically feasible is reached using this strategy.

### Sensing upon exposure to recombinant RBD

**Fig. 3:**
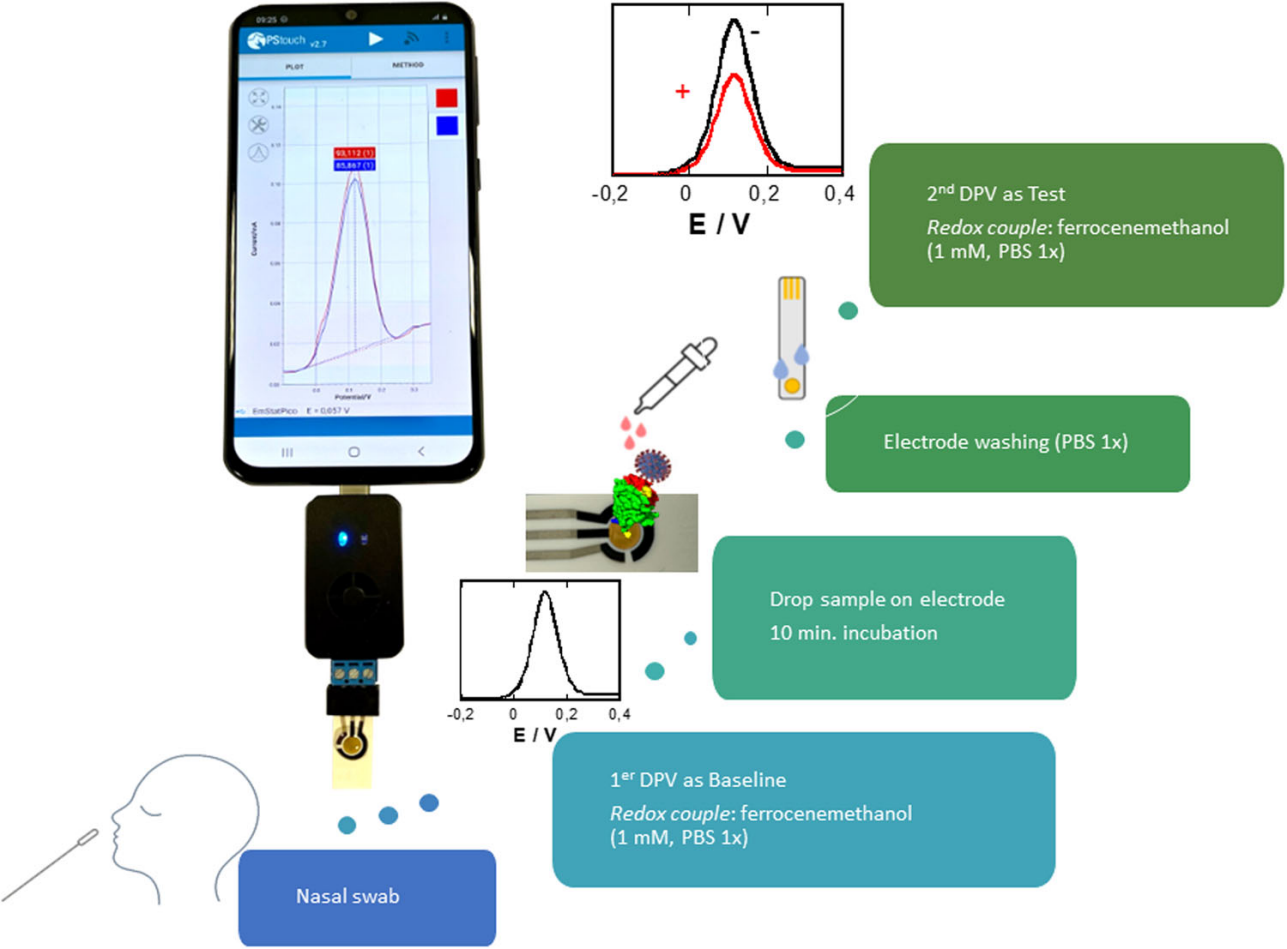
Nanobody-based COVID-19 voltammetric sensor. Schematic of the sensor concept together with working principle of the sensor and solutions used. Incubation time of sample before test via differential pulse voltammogram using ferrocenemethanol (1 mL, PBS 1×) as a redox probe is 10 min.

With a signal off working principle (Fig. 3), the sensing flow is as follows: (i) sample collection in universal transport medium (UTM), (ii) running a differential pulse voltammogram (DPV) using ferrocenemethanol (1 mL, PBS 1×) as a redox probe to record the initial signal, used as baseline to be compared to patient samples, (iii) incubation of the electrode in the patient sample (200 µL) for 10 min, (iv) washing electrode with PBS (1×), and (v) immersion into ferrocenemethanol (1 mM, PBS 1×) followed by recording of a DPV. The difference in the maximal current before and after sample interactions is used as positive or negative criteria.

**Fig. 4:**
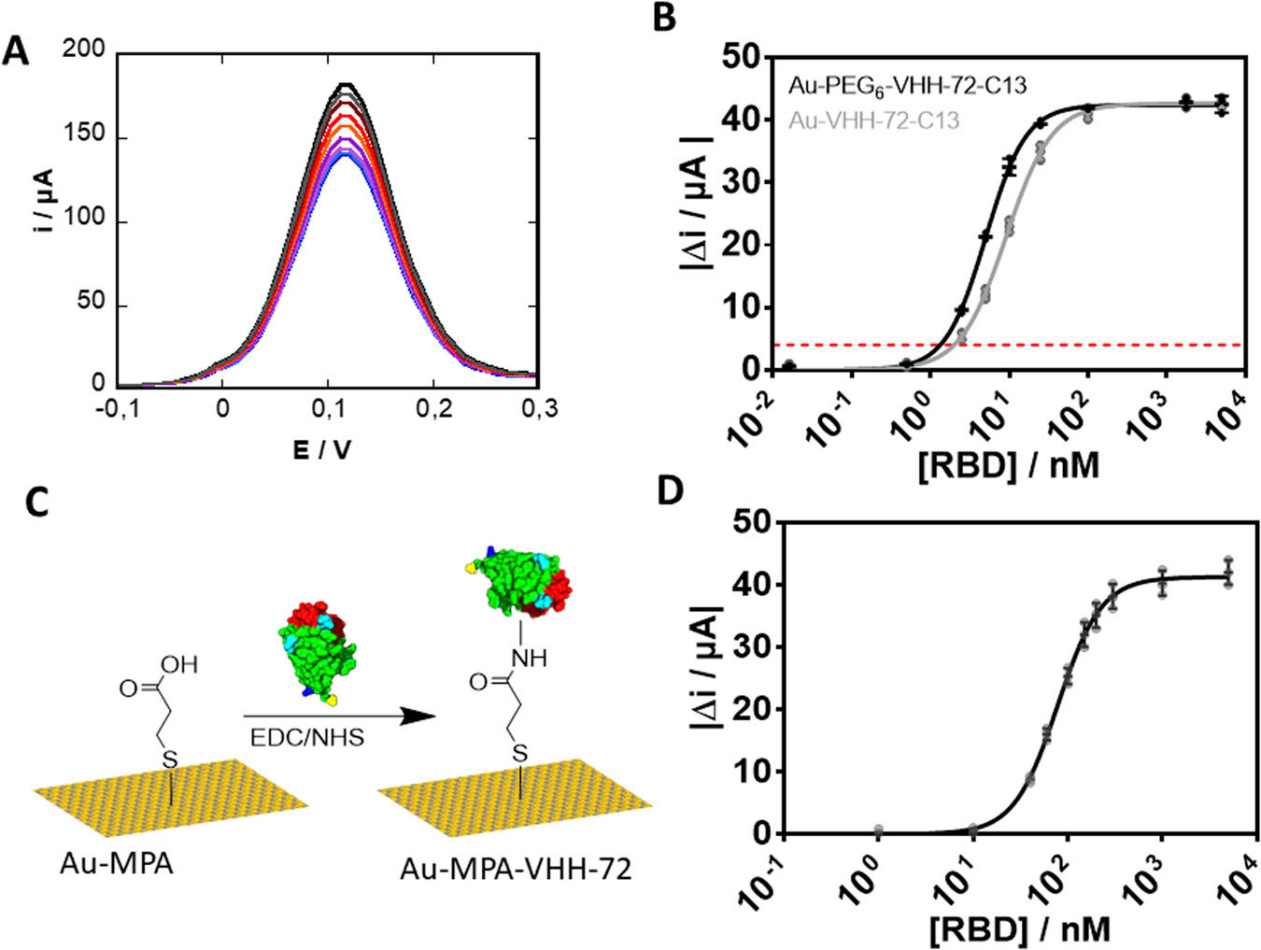
Performance of Au-VHH-72-C13, Au-PEG_6_-VHH-72-C13, and Au-MPA-VHH-72 electrodes. **A** Differential pulse voltammograms (DPVs) of Au-PEG_6_-VHH-72-C13 in ferrocenemethanol (1 mM in 0.1 M PBS, pH 7.4) initially (black) and after addition of RBD (1.2, 2.5, 5, 10, 25, 100, 1770, 5000 nM). **B** Dose–response curve of RBD of Au-PEG_6_-VHH-C13 as well as Au-VHH-C13 electrodes. Data were fitted to a Langmuir adsorption isotherm assuming a 1:1 complex between the antigen (RBD) from the solution and the linked VHH-72 receptor. **C** Surface attachment strategy of VHH onto 3-mercaptopropionic acid-modified gold (Au-MPA) interfaces using EDC/NHS coupling chemistry (protein, linkers, and gold surface are not drawn to scale with respect to each other). **D** Dose–dependent response curve of Au-MPA-VHH electrode to increasing RBD concentrations. The results are expressed as the mean ± SEM of at least 3 independent samples for each group.

The receptor binding domain (RBD) is a small (15 kDa) protein domain of the larger Spike 1 (S1) subunit and is directly responsible for recognition and interaction with host cell receptor. The sensing capability of Au-VHH-72-C13 and Au-PEG_6_-VHH-72-C13 electrodes for RBD is a good indication for their ability to sense SARS-CoV-2 viral particles. Exposure of Au-PEG_6_-VHH-72-C13 electrodes (Fig. 4A) to increasing RBD concentrations results in a decrease of the current density as the heterogeneous electron transfer at a VHH-72-C13 (estimated layer thickness 4 nm) and PEG_6_-VHH-72-C13 modified electrode (estimated layer thickness 5 nm) is strongly dependent on the thickness of the functionalization layer and the surface coverage. Interaction of RBD with the receptor domain of VHH translates in a decrease in the apparent rate constant for the redox reaction at hand. These observations are a result of optimized sensing parameters (E_step_ and t_pulse_), the quantity of nanobody used for surface attachment and reaction time, with 100 µg mL^−1^ of VHH-72-C13 and a thiol-maleimide interaction time for 2 h resulting in the most favourable RBD titration experiments.

From the dose–response curve (Fig. 4B), a limit of detection, defined as LoD = (3 × Noise)/Sensitivity, of 1.3 ± 0.6 nM was obtained using an estimated noise level of 4 µA and a Δi/ΔC_RBD_ determined as 9.4 µA nM^−1^. The S-shaped dose–response curve (Fig. 4B) can be fitted to a Langmuir isotherm assuming a 1:1 complex between the antigen (RBD) from solution and the linked VHH-72-C13 receptor, with a half saturation point (e.g., analyte concentration to occupy 50% of the receptor) of 3.7 ± 2.3 nM for Au-PEG_6_-VHH-72-C13 electrodes and 7.5 ± 1.7 nM for Au-VHH-72-C13. The improved orientation of VHH-72-C13 on Au-PEG_6_-VHH via the flexibility brought by the PEG units might be the underlying reason for the observed difference. In a control experiment, VHH-72 was covalently linked to Au-MPA (Fig. 4C) via the presence of lysine function in the nanobody rather than the cysteine end group using classical EDC/NHS coupling chemistry. The DPV recorded on Au-MPA-VHH-72 electrodes using ferrocenemethanol as a redox mediator displays comparable electrochemical signals as for Au-VHH-C13 and PEG_6_-VHH-C13 electrodes (Supplementary Fig. S4) with a current comparable to that of the PEG_6_-VHH-C13 interface, indicating comparable surface coverage and density of VHH-72. From the dose–response curve (Fig. 4D), an increased K_D_ value of 78 nM was determined, resulting in a LoD of 21 nM. The increased LoD and K_D_ values are related to the more random orientation of the nanobodies on the electrode surface with decreased RBD sensing capability.

The long-term stability of the sensor showed a loss of 4% when tested with RBD (100 nM) upon storage of the electrode at 4 °C for 30 days. Measurements at different temperatures (25 and 37 °C) did not alter the performance of the sensor.

### Electrochemical detection of SARS-CoV-2 virus in cultured virus samples

**Fig. 5:**
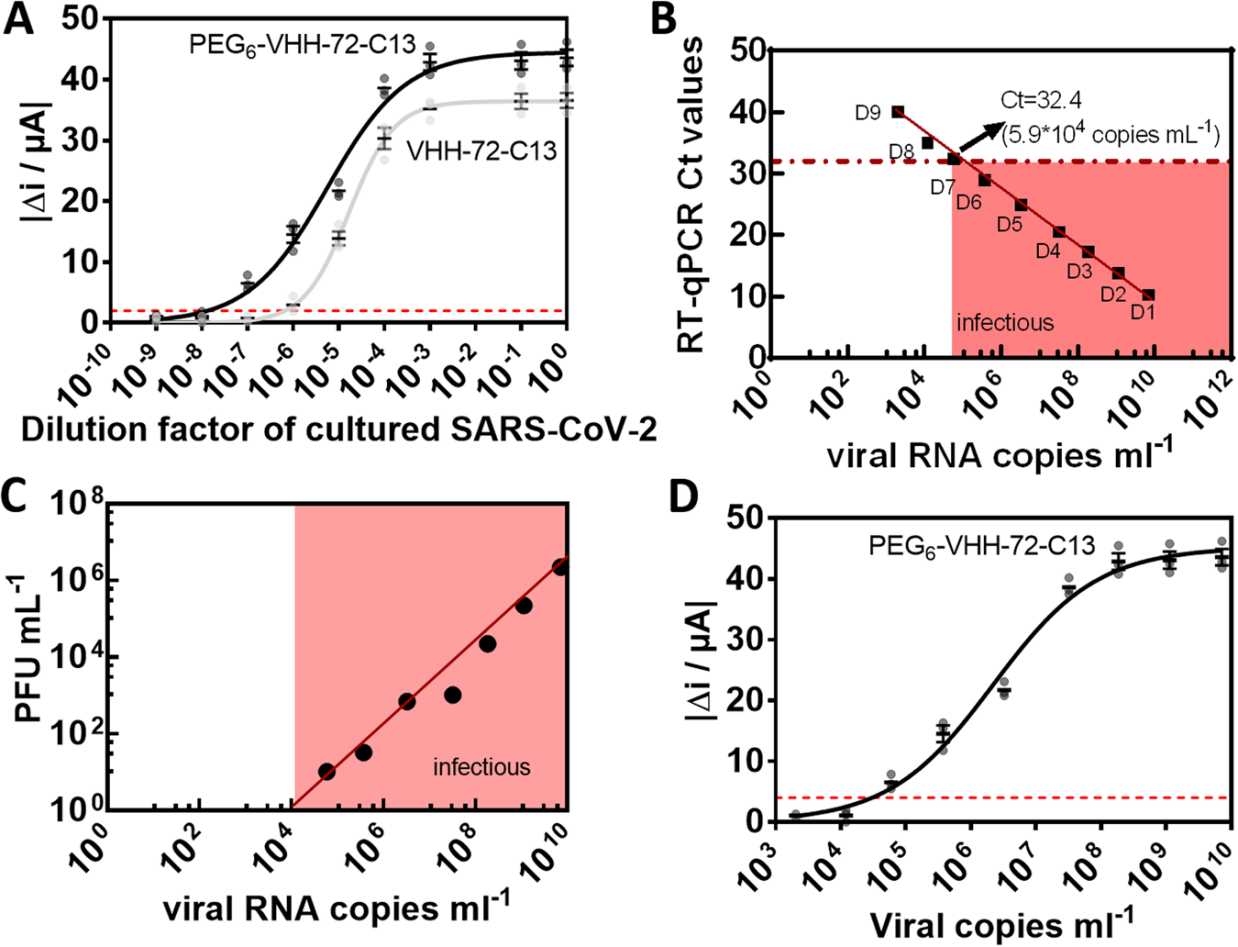
Performance of Au-PEG_6_-VHH-72-C13 and Au-VHH-72-C13 electrodes on cultured SARS-CoV-2 EU variant. **A** Dose-dependent response curves of 10-fold dilutions (D1-D9) of SARS-CoV-2, clade 20 A.EU2 (EU variant) on VHH-72-13C modified electrodes and VHH-72 for comparison. **B** Correlation of qRT-PCR Ct values and viral RNA copies mL^−1^. **C** Correlation of viral RNA copies mL^−1^ with plaque forming units of SARS-CoV-2 as a measure of infectivity. For this, Vero E6 cells were infected with 10-fold dilutions of a SARS-CoV-2 isolate clade 20 A.EU2 (EU variant). Calculation of estimated virus concentration was carried out by the Spearman and Karber method^20,21^ and expressed as TCID_50_/mL (50% tissue culture infectious dose). TCID_50_/mL values were transformed to PFU mL^−1^ by using the formula PFU mL^−1^ = TCID_50_/mL × 0.7 (https://www.lgcstandards-atcc.org/support/faqs/48802/Converting%20TCID50%20to%20plaque%20forming%20units%20PFU-124.aspx). RNA extraction and qRT-PCR (target IP4) were performed in duplicate for each dilution. **D** Dose-dependent response curves of viral copies of SARS-CoV-2, clade 20 A.EU2 (EU variant) on VHH-72-13C modified electrodes. The results are expressed as the mean ± SEM of at least 3 independent electrodes for each group.

After confirmation of the protein targeting possibility, we investigated the sensor performance on cultured SARS-CoV-2 clade 20 A.EU2 (EU variant) via viral dilution experiments (Fig. 5A). Clearly, the presence of the PEG_6_ ligand increased the detection capability of the sensor. To correlate the change in current to the absolute number of virions, the number of viral RNA copies was determined in parallel by qRT-PCR (Fig. 5B). As expected, a linear relation between RT-qPCR Ct values and viral RNA copies mL^−1^ (and thus dilution D1-D9) was observed. Using PEG_6_-VHH-72-C13 modified electrodes, the current difference of dilution 7 (D7) is clearly distinguishable from the background corresponding to 5.9 × 10^4^ copies, assuming that each genome is associated with one virion.

From Fig. 5A, a limit of detection (LOD), defined as LoD = (3 × Noise)/Sensitivity, of 1.2 × 10^4^ viral copies mL^−1^ is determined. A large fraction of viral RNA copies does not represent infectious viral particles. Therefore, the use of viral RNA copies as an approximation for the number of infectious viral particles leads to an overestimation. We, therefore, determined the infectious virus by determining the TCID_50_ (e.g., the 50% tissue culture infective dose) and consequently the number of plaque-forming units (PFU) with 1 PFU mL^−1^ = TCID_50_/mL × 0.7. Figure 5C indicates that 5.9 × 10^4^ copies mL^−1^ correlate to 10.3 ± 1 PFU mL^−1^. The Au-VHH-72-C13 and Au-PEG_6_-VHH-72-C13 sensors display a limit of detection of 2 ± 1 PFU mL^−1^ (Au-PEG_6_-VHH-72-C13) (equivalent to 1.2 × 10^4^ viral copies mL^−1^) and 30 ± 3 PFU mL^−1^ (Au-VHH-72-C13). With an infectivity cut-off value of SARS-CoV-2 for clade 20 A.EU2 (EU variant) of Ct = 32.4, the sensor performance is competitive with RT-qPCR.

**Fig. 6:**
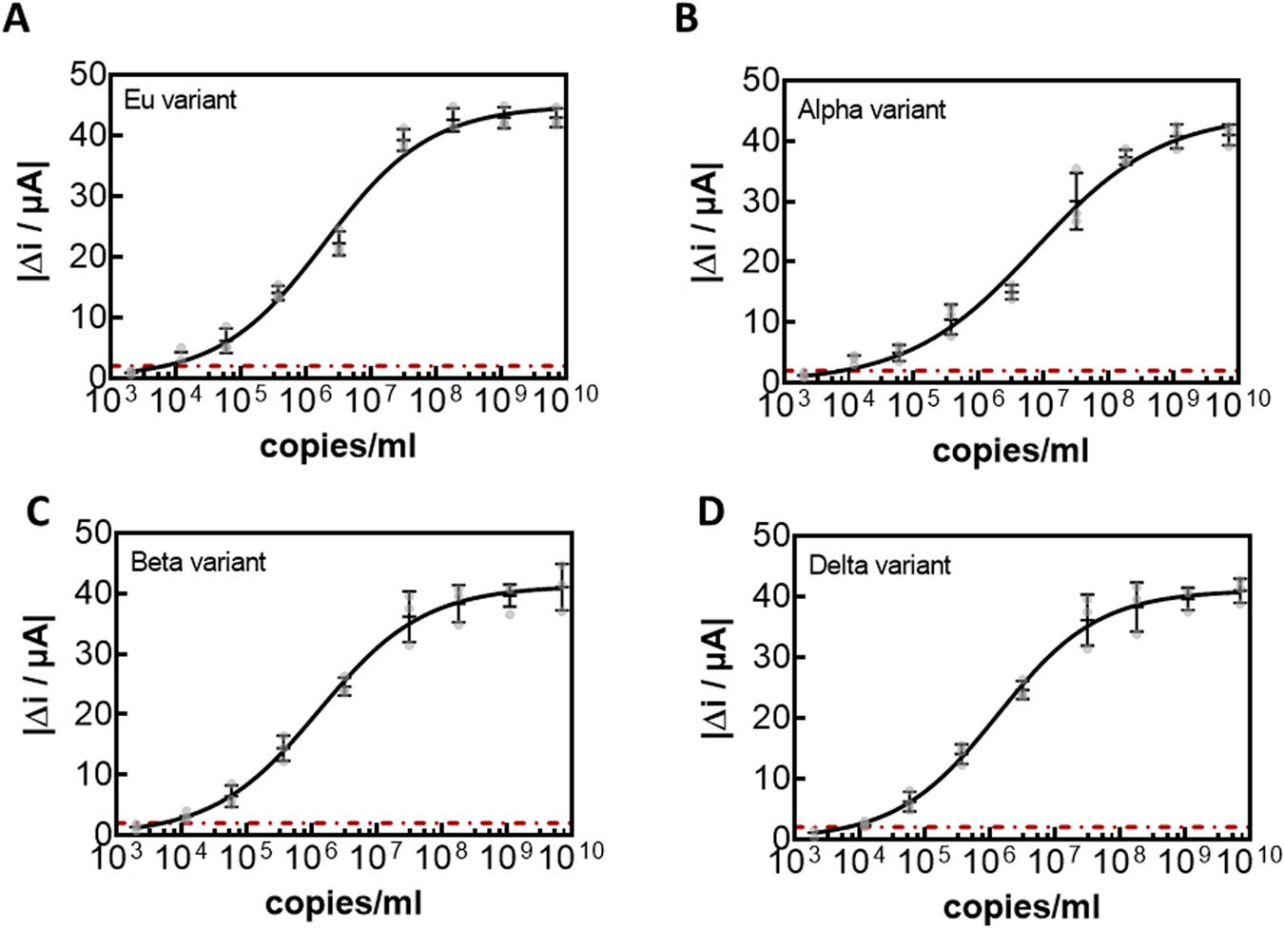
Dose-dependent response curves of Au-PEG_6_-VHH-72-C13 electrode for different SARS-CoV-2 cultured clades. **A** clade 20 A.EU2 (EU variant). **B** 20I/501Y.V1 (Alpha variant). **C** clade 20H/501Y.V2 (Beta variant). **D** B.1.617.2+AY.1+AY.2 (Delta variant). All results are expressed as the mean ± SEM of at least 3 independent electrodes for each group.

The risk with the direct detection of the virion via its surface protein is the occurrence of mutants in the RBD that could interfere with the nanobody epitopes. Recently, several mutants have been described in the RBD. In addition to the possibility of detecting clade 20 A.EU2 (EU variant) (Fig. 6A), variants 20I/501Y.V1 (Alpha variant) (Fig. 6B), clade 20H/501Y.V2 (Beta variant) (Fig. 6C), as well as B.1.617.2+AY.1+AY.2 (Delta variant) (Fig. 6D) were titrated with Au-PEG_6_-VHH-72-C13 electrodes. The correlation between viral copies mL^−1^ is seen in Supplementary Fig. S5 and used for correlation current measured due to virions present in the sample. The electrochemical sensor can sense the different variants with no significant difference (Fig. 6), indicating that the VHH-PEG_6_-72-C13 modified electrode can be rapid and able to detect any mutant.

We further explored the possibility to improve the sensor with the use of nanobody H11D4-13C specific for SARS-CoV-2 Spike RBD, displaying an improved K_D_^32^. For orientated surface immobilization, the cysteine-modified analogue, VHH-H11D4-13C, was produced and immobilized using the validated maleimide-PEG linkage strategy. Using electrochemical titration of RBD on the VHH-H11D4-13C modified gold electrode (Supplementary Fig. S6) resulted in a half saturation point at 3.4 ± 1.7 nM with a LOD for RBD of 3.2 ± 1.1 nM and a LoD of 7 ± 2 PFU mL^−1^ for clade 20 A.EU2 (EU variant). The lower half-saturation point of the VHH-H11D4-13C does not translate in a better LoD for RBD, neither in a better sensing performance of infective viral particles, which remained three times larger than for Au-PEG_6_-VHH, using VHH-72-C13 ligand.

### Sensor performance in clinical samples

**Fig. 7:**
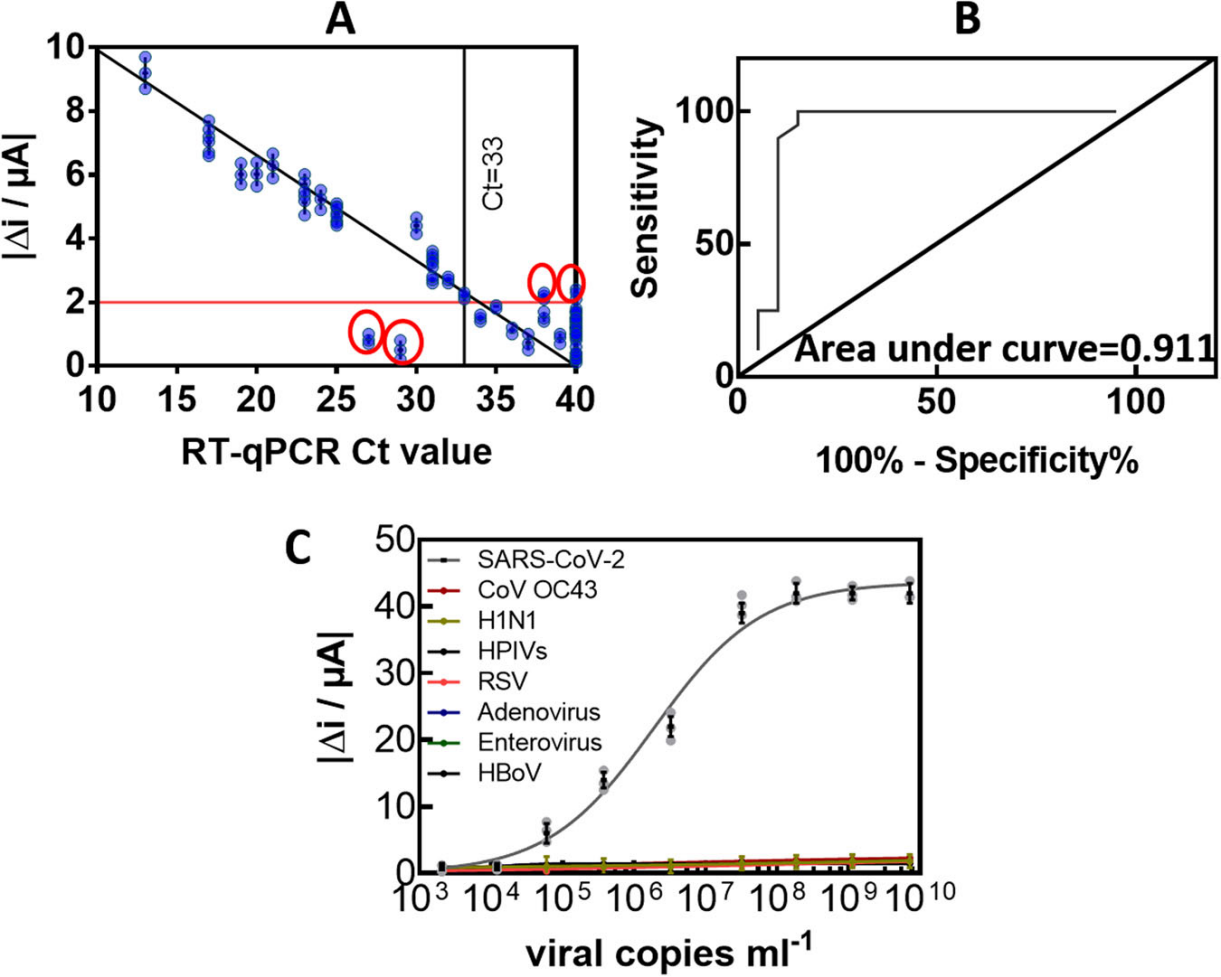
Performance of Au-PEG_6_-VHH-72-C13 sensor on clinical samples. **A** Current response difference for nasopharyngeal swab samples from COVID-19 positive patients (*n* = 20) and COVID-19 negative patients (*n* = 20) against the golden standard, RT-PCR Ct values. Cut-off level of positivity was set at Ct = 33. Cycle threshold value of RT-PCR (grey). The current cut-off value for positive samples was set to 2 µA. Each patient sample was performed on a new electrode. The results are expressed as the mean ± SEM of 3 independent measurements for each sample. **B** ROC curve from clinical data and positive and negative RT-PCR identification. **C** Selectivity of the SARS-CoV-2 electrochemical diagnostics towards other viruses. The results are expressed as the mean ± SEM of 3 independent measurements for each sample.

The performance of the Au-PEG_6_-VHH sensor to discriminate between SARS-CoV-2 positive and negative nasopharyngeal swab samples was evaluated and collected from patients in a clinical testing facility. Swabs were received in universal transport medium (UTM) and 200 µL of this sample was analysed immediately after sampling. The interaction time between the viral samples and the sensing surface was set to 10 min, as longer interaction times did not show improved results. From the 20-nasal swab (Fig. 7A) samples that had been confirmed by RT-qPCR to be positive, 18 were identified as COVID-19 positive with the Au-PEG_6_-VHH sensor; an accordance with RT-qPCR of 90% positive percentage agreement (PPA). To see if quantitative analysis is reached with the sensor concept, the patient samples were ordered from high viral loads (Ct = 13) to lower viral loads (Ct = 40). Figure 7A indicates that indeed lower Ct values correlate with higher current changes. Close to the infectivity limit, current differences of 2.5 µA are detected, being still above the threshold of 2 µA, defined as the noise level of the detection. In a second validation, 20 nasal swab samples confirmed as negative by RT-PCR were analysed with the electrochemical sensor. Out of the 20 negative samples, 18 were identified correctly with a 90% negative percentage agreement (NPA) resulting in a sensor of 90% sensitivity and 90% specificity via the gold standard RT-qPCR. A receiver operating characteristic (ROC) curve was calculated (Fig. 7B) by using the electrochemical measurements of clinical specimens with 20 SARS-CoV-2 RT-PCR positive specimens (Ct value ≤33) and 20 specimens with Ct values higher than 33 (defined as negative). The area under the curve was 0.911.

The analysis of the two-medical charts of the patients, diagnosed negative in RT-qPCR and positive with our diagnostic technology, revealed that these persons had a notion of contact with a COVID-19 positive person and a compatible clinical presentation including hyperthermia cough, rhinorrhoea, expectoration and hyposmia. Since the presentation was very compatible, serology was performed but too early to conclude and was not repeated. Thus, the final diagnosis remained questionable. It can be suggested that these patients could have been correctly identified by our technology.

In the case of the two cases diagnosed negative with the sensor but positive with RT-qPCR, one person was totally asymptomatic, but showed positive PCR, while no positive serology results were obtained. This person could be considered false positive by RT-qPCR and the results of the sensor might not be really wrong. The other person had a notion of COVID-19 positive contact and compatible clinical presentation. This is considered a true COVID-19 positive and the sensor did not detect this patient.

These data show that the developed diagnostic test technique that has a high level of sensitivity and specificity due to its concordance of 90% with the reference RT-qPCR test, whose relevance is not in doubt. Some of the current antigenic tests on the market have similar sensitivity; yet it is another promising approach with lots of potential.

The sensor was tested for its selectivity for 5 other viruses (Fig. 7): (i) Human coronavirus OC43 (HCoV-OC43), a member of the betacoronavirus 1 species producing symptoms close to those associated SARS-CoV-2. (ii) Influenza A(N1H1) pdm09 virus (pH1N1), the causal factor of the recent flu pandemic. (iii) Human parainfluenza virus (HPIVs) a common respiratory tract pathogen that can infect persons of any age. (iv) Human bocavirus (HBoV) a member of the Parvoviridae virus family that are small (20 nm), non-enveloped viruses with single-stranded DNA found usually in infants and children who are hospitalized with pneumonia or diarrheal symptoms. (v) Respiratory syncytial (sin-SISH-uhl) virus, or RSV, is a common respiratory virus that usually causes mild, cold-like symptoms. (vi) a human adenovirus, a medium-sized (90–100 nm), nonenveloped virus with an icosahedral nucleocapsid containing a double stranded DNA genome and (vii) Enterovirus 71, a positive-sense single-stranded RNA viruses associated with several human and mammalian diseases. The detection of classical respiratory viruses is routinely performed using commercial multiplex RT-qPCR assay and all of these samples were considered as positive. Discrimination with influenza A(N1H1) pdm09 and human parainfluenza virus (HPIVs) is insured. Nasal swab samples of the other human coronavirus OC43 (HCoV-OC43) of high and low viral loads showed in addition not cross-reactivity with our sensor.

**Fig. 8:**
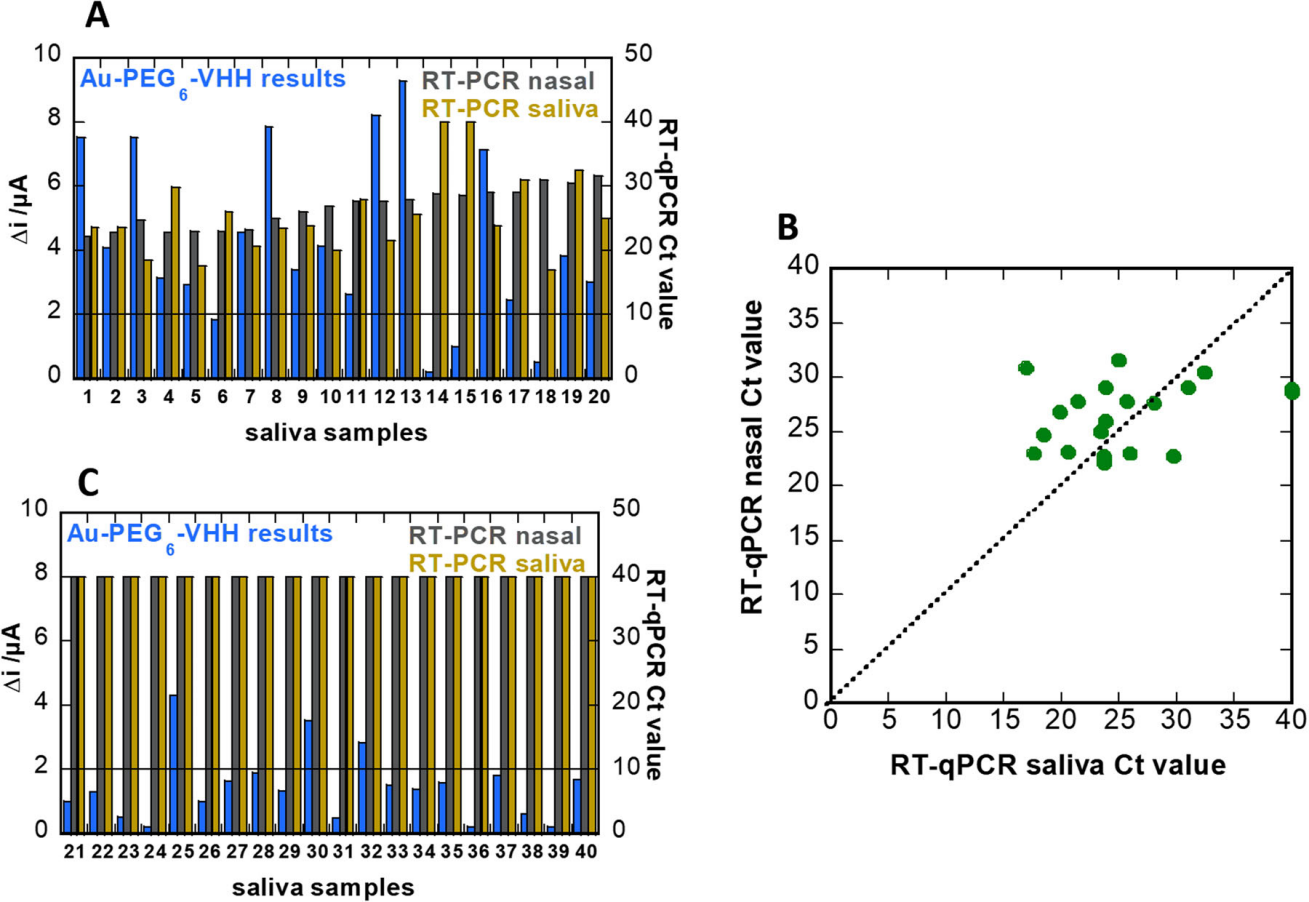
Performance of sensor in saliva clinical samples. **A** Response diagram for saliva samples from COVID-19 positive patients (*n* = 20) as recorded on Au-PEG_6_-VHH electrodes (blue). Cycle threshold value of RT-qPCR on nasal swab samples of same patient (grey), as well as RT-PCR of saliva (brown). The current cut-off value for positive samples was 2 µA. **B** Collection of RT-qPCR values of nasopharyngeal swab samples vs. saliva samples collected from the same patient and measured in the hour after sample taking. **C** Response diagram for saliva samples from COVID-19 negative patients (*n* = 20) as recorded on Au-PEG_6_-VHH electrodes (blue) and Cycle threshold value of RT-PCR on nasal swab samples (grey), and saliva (brown).

For further validation, we collected saliva samples from 20 healthy and 20 COVID-19 positive patients. Each sample was diluted one-fold in PBS (0.1 M) and filtered through a desalination column (Sephadex® G-25 packed,) using gravity for separation, to filter large proteins, such as proteases and amylase. In parallel, 20 nasopharyngeal samples were collected from the same patients and quantified by RT-qPCR. Despite substantial variation in the individual sensor readings, the sensor agreed with PCR results of nasal swab samples with 80% agreement (Fig. 8A). Analysis of the Au-PEG_6_-VHH electrodes revealed 16 out of 20 samples as positive when compared to PCR of nasal swab samples. Interestingly, when comparing these samples (samples 14 and 15) to PCR of saliva samples, they agreed with the electrochemical sensing signal. That might indicate that indeed no virus was present in the saliva samples. Indeed, from the correlation of RT-qPCR results of nasal swab samples and saliva samples collected from the same patients at the same time (Fig. 8B), only a 90% concordance was observed.

However, in contrast with results of nasopharyngeal samples (Fig. 7), there is no direct correlation between the current signal and the viral loads, as established by RT-qPCR. The sensor response might be falsified by the presence of saliva proteases and other saliva proteins, even in the presence of PEG as anti-fouling interface. In the case of the 20 negative saliva samples, the agreement with the electrochemical diagnostics is 85% (17 out of 20) (Fig. 8C).

## Conclusions

The gold standard for COVID-19 diagnostics relies on RT-PCR of nasal swab samples that needs trained personnel, and is limited in its usability for rapid diagnostic in a point-of-care testing format. Thus, efforts are being put into developing rapid tests based on technologies accessible to the population at large. The most effective strategy widely adopted is based on the detection of the presence of SARS-CoV-2 antigens such as the S1 Spike envelop protein and/or virus specific N protein. These point-of-care assays are typically based on an immunosensing strategy based on S1 and N protein specific antibodies. This work describes a nanobody-based electrochemical sensor capable to specifically detect SARS-CoV-2 via the S1 spike in nasopharyngeal patients’ samples with a 90% PPA and 90% NPA concordance to RT-PCR with a used Ct cut off of 34 (i.e., representing the sensitivity and specificity since RT-PCR is the gold standard). The importance of a controlled surface orientation of the nanobody receptor as well as the implementation of an anti-fouling strategy has been the underlining success of this work. We showed that the device sensitivity competes well with the RT-PCR detection method using VHH-72-13C, and higher affinity ligands such as VHH-11HD4-13C have no relevant impact on the sensing ability. This work opens up the possibility of point-of-care detection of SARS-CoV-2 infection due to its acceptable sensitivity and could add to the current COVID-19 diagnosis workflows. Such an efficient approach would reduce the risk of viral spreading due to the possibility of immediate isolation in case of positivity. The approach we describe here provides also a cost-effective solution to the crisis as it does not require trained people to perform the test once samples were collected. The only risk with the direct detection of the virion via its surface protein is the occurrence of mutants in the RBD that could interfere with the nanobody epitopes. Thanks to the sensitivity of our device and the selected ligand, this issue has been overcome by testing several SARS-CoV-2 mutants. In conclusion, although there is room to easily improve our device and adapt it to new pandemic developments, we already have an efficient tool which provides results in a short time, is portable, allows direct use in emergency or consulting rooms, and can be performed easily. The electrodes are currently single use devices, with costs in the same order as for current antigenic test. In a later stage, the implementation of wireless data transmission of the results in real time to the biological teams makes the approach a real point-of-care device ensuring optimal medical care. The functionality of the test has still to be improved so that people can more easily use it for self-testing and work on the second generation of the nanobody-based electrochemical sensor is ongoing.

## Supplementary information

Supplementary Information

Supplementary Data 1

Reporting Summary

Description of Additional Supplementary Files

## Data Availability

The datasets generated during the current study are available from the corresponding author on reasonable request. Source data for the main figures can be assessed as Supplementary Data 1.
