# Editorial Expression of Concern: SARS-CoV-2 detection using a nanobody-functionalized voltammetric device

**DOI:** 10.1038/s43856-026-01399-8

**Published:** 2026-02-02

**Authors:** Quentin Pagneux, Alain Roussel, Hiba Saada, Christian Cambillau, Béatrice Amigues, Vincent Delauzun, Ilka Engelmann, Enagnon Kazali Alidjinou, Judith Ogiez, Anne Sophie Rolland, Emmanuel Faure, Julien Poissy, Alain Duhamel, Rabah Boukherroub, David Devos, Sabine Szunerits

**Affiliations:** 1 Univ. Lille, CNRS, Centrale Lille, Univ. Polytechnique Hauts-de-France UMR 8520 - IEMN, Lille, France; 2 Laboratoire d’Ingénierie des Systèmes Macromoléculaires (LISM), Institut de Microbiologie, Bioénergies et Biotechnologie (IM2B), Aix-Marseille Université - CNRS UMR, Marseille, France; 3 Univ Lille, CHU Lille, Laboratoire de Virologie, Lille, France; 4 Univ. Lille, CHU-Lille, Inserm, U1172, Lille Neuroscience & Cognition, LICEND, Lille, France; 5 Service Universitaire de maladies infectieuses - Hôpital Hutiez, CHU de Lille, Lille, France; 6 UMR8204 U1019, Centre infection et immunité de Lille, Equipe Opinfield, Institut Pasteur de Lille, Lille, France; 7 Univ. Lille, Inserm U1285, CHU Lille, Pôle de réanimation, CNRS, UMR 8576 - UGSF - Unité de Glycobiologie Structurale et Fonctionnelle, Lille, France; 8 Univ. Lille, CHU Lille, ULR2694 METRICS: évaluation des technologies de santé et des pratiques médicales, Lille, France

Editorial Expression of Concern: *Communications Medicine* 10.1038/s43856-022-00113-8; published online 23 May 2022

The Editor would like to alert the readers that concerns have been raised regarding some of the data presented in this article and its supplementary materials, including low data variability and discrepancies between the plots presented in the Figures and the source data, specifically:

- The data presented in Fig. 2E for Au-PEG_6_-VHH-72-C13 and Fig. 4A 1.2 nM RBD as well as the associated values in the source data files appear highly similar;
- The data presented in Figs. 2E and 4A appear to have unusually low variability between groups;
- The data presented in Figs. 5D, 6C, 6D and 7C appear to differ from the source data.

The authors have stated that there were errors in the source data files for Fig. 5D and 7C, and that Fig. 6C incorrectly included data of a second Delta variant instead of the Beta variant.

Due to the number of errors, readers are advised to interpret these data with caution.

The authors have not responded to any correspondence from the editor or publisher about this Editorial Expression of Concern.

