# Supplementary material for: SARS-CoV-2 detection using a nanobody-functionalized voltammetric device
Source: Commun Med (Lond). 2022 May 23;2:56. doi: 10.1038/s43856-022-00113-8 (PMC9126950; doi:10.1038/s43856-022-00113-8)
Supplement: Supplementary file 1 — Supplementary Information [file 43856_2022_113_MOESM1_ESM.pdf]

Supplementary Information

SARS-CoV-2 detection using a nanobody-functionalized voltammetric device

Quentin Pagneux,[1] Alain Roussel,[2*] Hiba Saada, [1] Christian Cambillau,[2] Béatrice Amigues,[2] Vincent Delauzun,[2] Ilka Engelmann,[3] Enagnon Kazali Alidjinou,[3] Judith Ogiez,[3] Anne Sophie Rolland,[4] Emmanuel Faure,[5,6] Julien Poissy,[7] Alain Duhamel,[8] Rabah Boukherroub,[1] David Devos,[4*] Sabine Szunerits[1*]

[1]*Univ. Lille, CNRS, Centrale Lille, Univ. Polytechnique Hauts-de-France, UMR 8520 - IEMN, F-59000 Lille, France*

[2]*Laboratoire d'Ingénierie des Systèmes Macromoléculaires (LISM), Institut de Microbiologie, Bioénergies et Biotechnologie (IM2B), Aix-Marseille Université - CNRS, UMR 7255, Marseille, France.*

[3] *Univ Lille, CHU Lille, Laboratoire de Virologie ULR3610, F-59000 Lille, France*

[4] *Univ. Lille, CHU-Lille, Inserm, U1172, Lille Neuroscience & Cognition, LICEND, Lille, France*

[5] *Service Universitaire de maladies infectieuses - Hôpital Hutiez, CHU de Lille, F-59000, Lille, France*

[6] *UMR8204 U1019, Centre infection et immunité de Lille, Equipe Opinfield, Institut Pasteur de Lille, F-59800, Lille, France*

[7] *Univ. Lille, Inserm U1285, CHU Lille, Pôle de réanimation, CNRS, UMR 8576 - UGSF - Unité de Glycobiologie Structurale et Fonctionnelle, F-59000 Lille, France*

[8] *Univ. Lille, CHU Lille, ULR2694 METRICS : évaluation des technologies de santé et des pratiques médicales, F 59000 Lille, France*

* Correspondence: alain.roussel@univ-amu.fr, David.Devos@chru-lille.fr, sabine.szunerits@univ-lille.fr

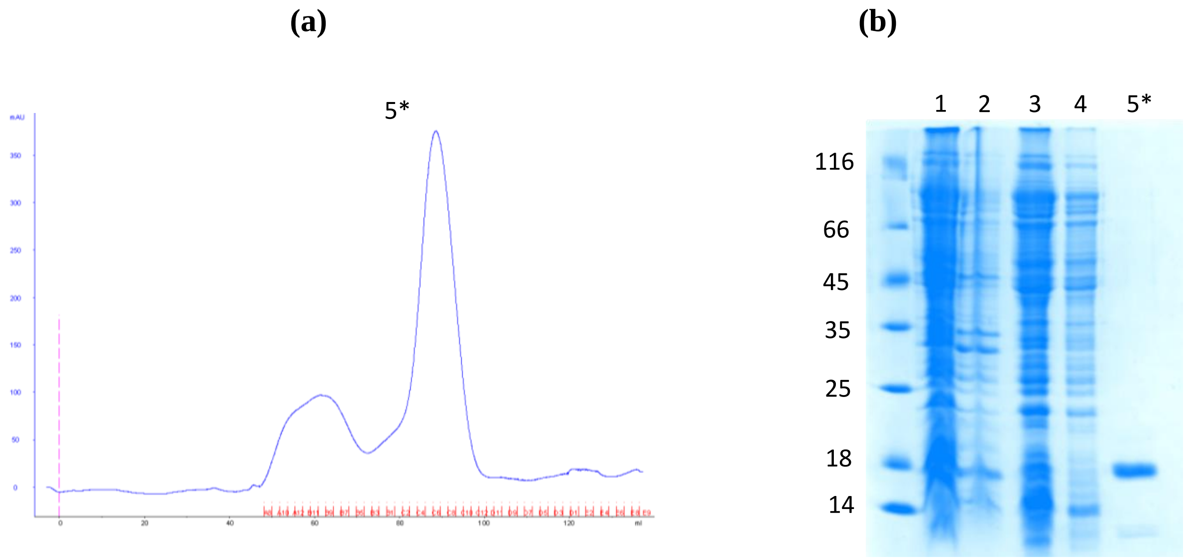

Figure S1. (a) Affinity and size exclusion chromatography: (b) SDS-PAGE, acrylamide 15 %, Coomassie blue staining: 1: Total. 2: Pellet. 3: Soluble fraction. 4: Nickel / Flow-through. 5: Gel filtration / Elution

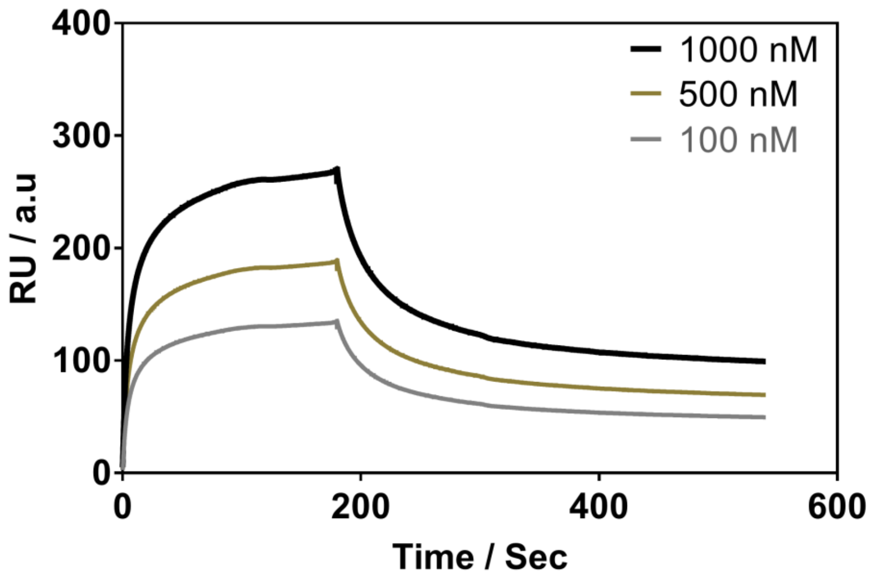

Figure S2. SPR binding curve of RBD to VHH-72 modified gold SPR chip (recorded on Biacore T200). The SPR binding corves were recorded on a Biocore T200 (Cytiva Life Science, using as running buffer HBS-P+ 1× (containing 0.1 M HEPES, 1.5 M NaCl and 0.5% v/v Surfactant P20. A CM5 SPR chip was employed and after activation using NHS/EDC (15 mM in HBS-P+ 1×) for 30 sec (flow rate= 5µL min^{-1}, VHH-72 (100 µg mL^{-1} in HBS-P+ 1×) was flown over surface for 30 sec at 5µL min^{-1} to reach a modification response of 200 RU (equal to 0.2 ng mm^{-2}). The interaction with RBD (in HBS-P+) was at a flow of 30µL min^{-1}.

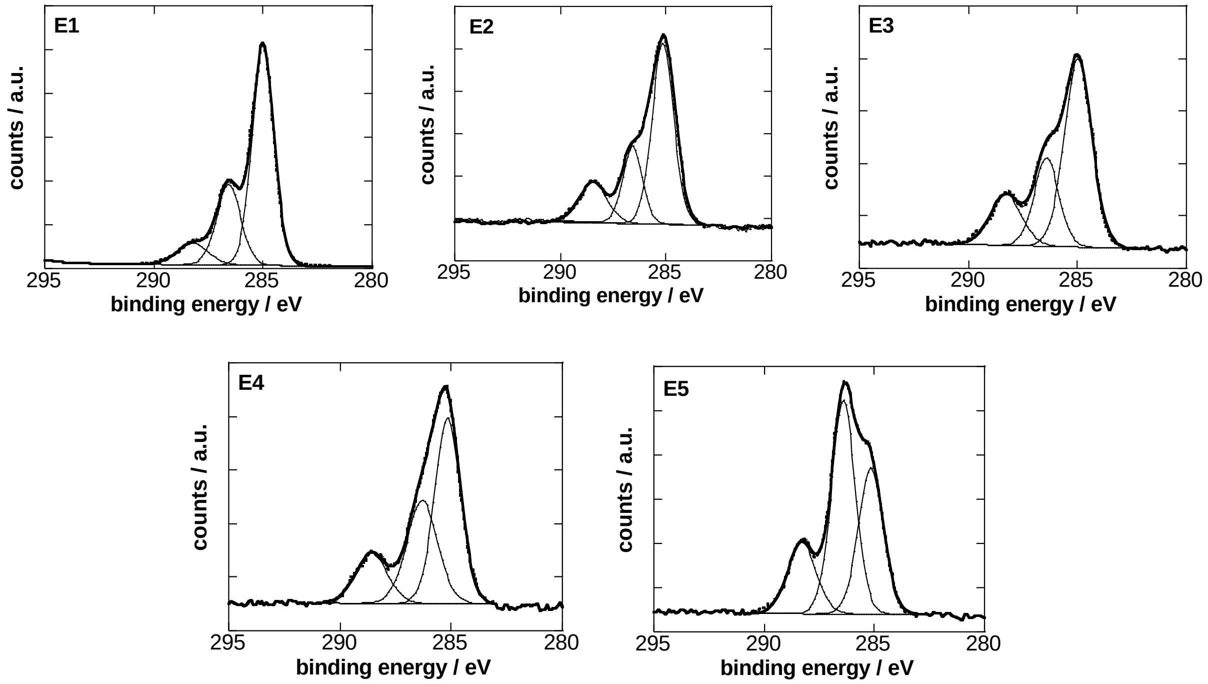

Figure S3. **High resolution of the C1s region of interfaces E1-E5** (**Table 1**). The gold surface was modified with 3-mercaptopropinic acid before (**E1**) and after (**E2**) VHH-72 immobilization. (E3) Direct linkage of VHH-72. Gold electrode modified with 3-mercaptopropinic acid, followed by NH_2-PEG-maleimide linkage before (**E4**) and after (**E5**) VHH-72-13C as immobilization.

Table S1. Element percentage obtained from XPS analysis for electrodes E1-E5

		**Atomic percentage (%)**			
**Electrode**	**Modification strategy**	**C1s**	**O1s**	**N1s**	**S2p**
**E1**	3-Mercaptopropionic acid (MPA)	75.3±0.3	20.6±0.2	-	4.1±0.5
**E2**	1. MPA<br>2. VHH-72	69.8±0.5	18.9±0.4	8.9±0.2	2.4±0.5
**E3**	1. VHH-72-13C	69.9±0.4	20.2±0.2	8.8±0.4	1.1±0.2
**E4**	1. MPA<br>2. NH_2-PEG_6-maleimide	69.5±0.4	20.1±0.8	6.2±0.5	4.2±0.5
**E5**	1. 3-Mercaptopropionic acid<br>2. NH_2-PEG_6-maleimide<br>3. VHH-72-13C (ou VHH11D4-13C)	65.3±0.5	21.4±0.6	9.2±0.2	4.1±0.7

The success of the integration of VHH-72 was validated by XPS analysis. The C1s high resolution spectra of the 5 interfaces are seen in **Figure S3**. The C1s spectrum of **E1** can be deconvoluted into bands at 285.0 eV (C-C/C-H), 286.7 eV (C-S/C-O) and 288.2 (O-C=O) eV in accordance with the chemical composition of the electrode surface. The C1s high resolution spectrum of the surface **E2**, prepared by direct linking of VHH-72 *via* amide bond formation,

can be curve-fitted with several bands at 285.0 eV (C-C/C-H), 286.7 eV (C-N/C-S/C-O) and 288.6 eV (O-C=O/ N-C=O).

The C1s spectrum of interface **E3**, obtained through the direct linkage of VHH-C13 to gold surface, shows comparable features as E2, with bands at 285.0 eV (C-C/C-H), 286.7 eV (C-S/C-O/C-N) and 288.6 eV (O-C=O/N-C=O).

The core level XPS spectrum of the C1s of the pegylated gold interface functionalised with maleimide terminal group (**E4**) can be fitted with several components at 285.0 eV (C-C/C-H), 286.3 eV (C-O) and 288.7 eV (C=O and N-C=O), in full agreement with the chemical composition of the surface. Incorporation of VHH-72-C13 or VHH-11D4-13C *via* thiol-maleimide bond formation (**E5**) induces slight changes in the C1s band with contributions at 285.0 eV (C-C/C-H), 286.3 eV (C-O, C-N, C-S) and 288.2 eV (C=O). The increase of the band at 286.3 eV is due to the introduction of additional C-N and C-S bonds brought by the VHH-72-C13 or VHH-11D4-13C.

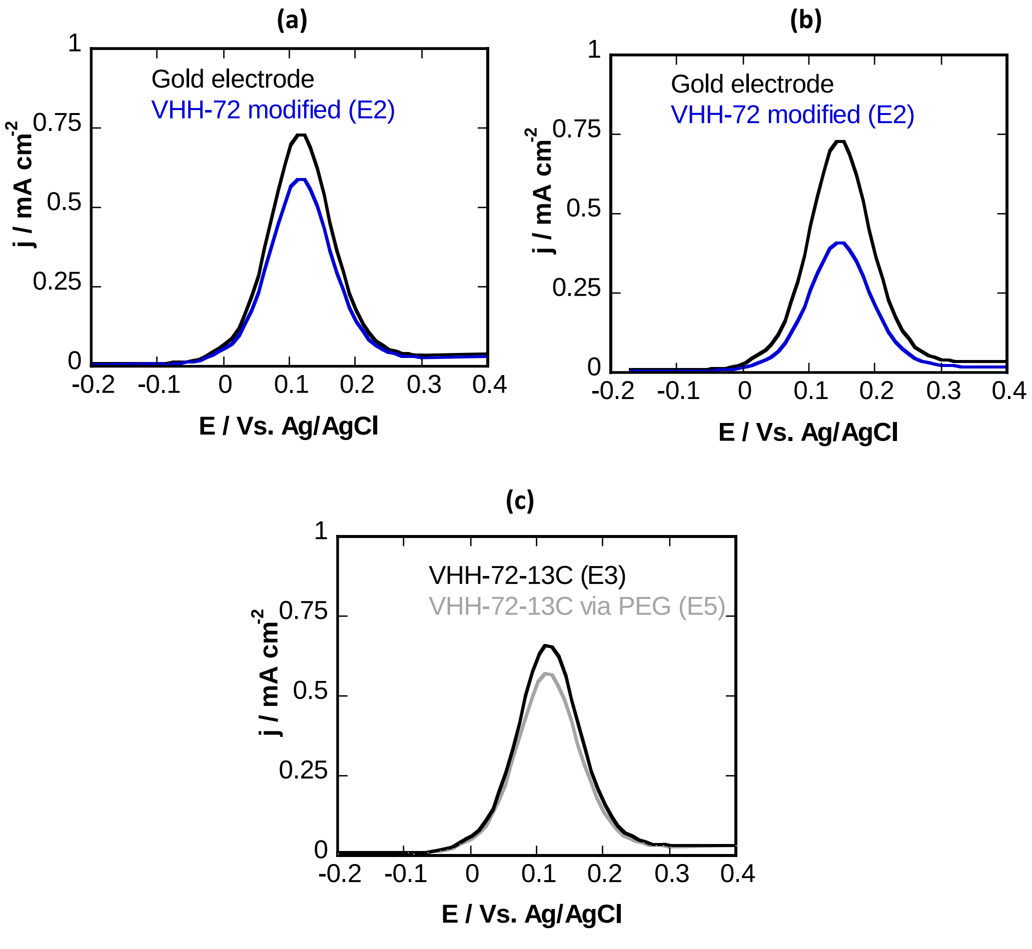

Figure S4. **Differential pulse voltammograms of different electrodes**: (a) Gold electrode before (black) and after (blue) VHH-72 immobilization (surface **E2**) in ferrocenemethanol (1 mM in 0.1 M PBS, pH 7.4). (b) Gold electrode before (black) and after (blue) VHH-72 (surface **E2**) immobilization in $Fe(CN)_6^{4-/3-}$ (1 mM in 0.1 M PBS, pH 7.4). (c) Gold electrode modified with VHH-72-13C (surface **E3**) and via maleimide-modified PEG linker (surface **E5**) immobilization in ferrocenemethanol (1 mM in 0.1 M PBS, pH 7.4).

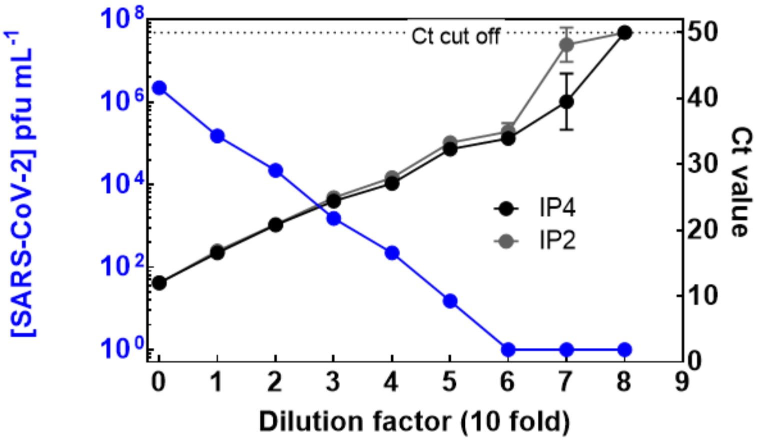

Figure S5: Correlation of Ct values with SARS-CoV-2 infectivity: Vero E6 cells (2.5×10^5 cells/well) were infected with 10-fold dilutions of a SARS-CoV-2 isolate. The plates were incubated for 6 days in 5% CO_2 atmosphere at 37 °C and examined daily using an inverted microscope (ZEISS Primovert) to evaluate the extent of the virus-induced cytopathic effect in cell culture. Calculation of estimated virus concentration was carried out by the Spearman and Karber method[1,2] and expressed as $TCID_{50}$/mL (50% tissue culture infectious dose). $TCID_{50}$/mL values were transformed to PFU mL^{-1} by using the formula PFU mL^{-1} = $TCID_{50}$/mL × 0.7 RNA extraction.[3] Negative RT-PCR results were set to Ct= 50. The results are expressed as the mean ± SEM of at least 4 independent samples for each group.

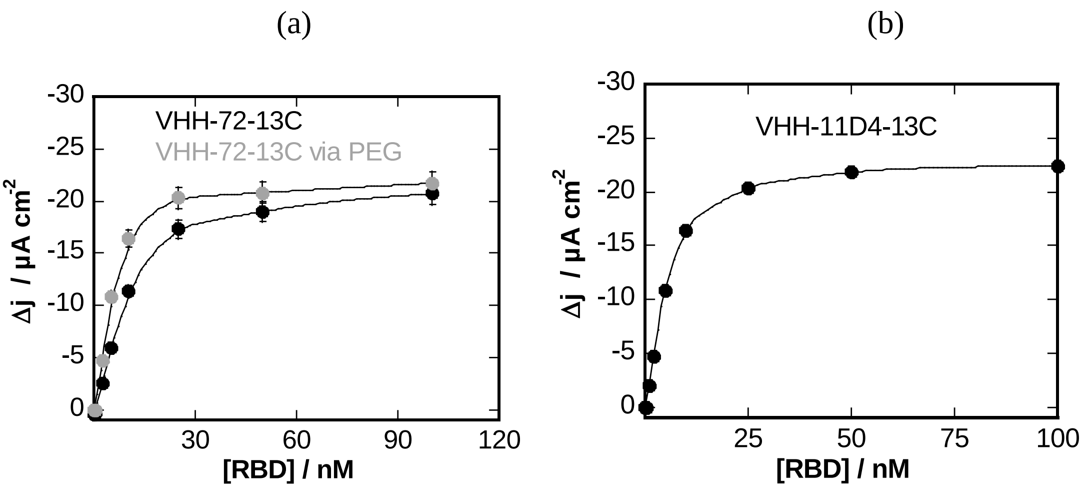

Figure S6: Calibration curves for RBD on VHH-72-13C and VHH11D4-13C modified interfaces. Change in current density responses towards RBD standard addition on gold electrodes modified with (a) VHH-72-C13 *via* direct linkage (surface **E3**) with a PEG linker (surface **E5**), and (b) CHH11D4-13C using ferrocenemethanol (1 mM in 0.1 M PBS, pH 7.4) as redox probe. The results are expressed as the mean ± SEM of at least 3 independent samples for each group.

Supplementary References

1 Spearman, C., The Method of "Right and Wrong Cases" (Constant Stimuli) without Gauss's Formula. Br. J. Psychol. 1908, 2, 227-242.

2 Kärber, G., Beitrag zur kollektiven Behandlung pharmakologischer Reihenversuche. . Arch. exp. Pathol. U. Pharmakol. 1931, 162, 480-483.

3 https://www.lgcstandards-atcc.org/support/faqs/48802/Converting%20TCID50%20to%20plaque%20forming%20units%20PFU-124.aspx.
