# Supplementary material for: SARS-CoV-2 detection using a nanobody-functionalized voltammetric device
Source: Commun Med (Lond). 2022 May 23;2:56. doi: 10.1038/s43856-022-00113-8 (PMC9126950; doi:10.1038/s43856-022-00113-8)
Supplement: Supplementary file 3 — Reporting Summary [file 43856_2022_113_MOESM3_ESM.pdf]

nature research

Corresponding author(s): Sabine Szunerits

Last updated by author(s): 20021-11-17

Reporting Summary

Nature Research wishes to improve the reproducibility of the work that we publish. This form provides structure for consistency and transparency in reporting. For further information on Nature Research policies, see our Editorial Policies and the Editorial Policy Checklist.

Statistics

For all statistical analyses, confirm that the following items are present in the figure legend, table legend, main text, or Methods section.

n/a	Confirmed	
☐	☒	The exact sample size (n) for each experimental group/condition, given as a discrete number and unit of measurement
☐	☒	A statement on whether measurements were taken from distinct samples or whether the same sample was measured repeatedly
☒	☐	The statistical test(s) used AND whether they are one- or two-sided<br>*Only common tests should be described solely by name; describe more complex techniques in the Methods section.*
☒	☐	A description of all covariates tested
☒	☐	A description of any assumptions or corrections, such as tests of normality and adjustment for multiple comparisons
☒	☐	A full description of the statistical parameters including central tendency (e.g. means) or other basic estimates (e.g. regression coefficient) AND variation (e.g. standard deviation) or associated estimates of uncertainty (e.g. confidence intervals)
☒	☐	For null hypothesis testing, the test statistic (e.g. F, t, r) with confidence intervals, effect sizes, degrees of freedom and P value noted<br>*Give P values as exact values whenever suitable.*
☒	☐	For Bayesian analysis, information on the choice of priors and Markov chain Monte Carlo settings
☒	☐	For hierarchical and complex designs, identification of the appropriate level for tests and full reporting of outcomes
☒	☐	Estimates of effect sizes (e.g. Cohen's d, Pearson's r), indicating how they were calculated

Our web collection on statistics for biologists contains articles on many of the points above.

Software and code

Policy information about availability of computer code

Data collection	no software was used
Data analysis	no software was used

For manuscripts utilizing custom algorithms or software that are central to the research but not yet described in published literature, software must be made available to editors and reviewers. We strongly encourage code deposition in a community repository (e.g. GitHub). See the Nature Research guidelines for submitting code & software for further information.

Data

Policy information about availability of data

All manuscripts must include a data availability statement. This statement should provide the following information, where applicable:

- Accession codes, unique identifiers, or web links for publicly available datasets
- A list of figures that have associated raw data
- A description of any restrictions on data availability

The datasets generated during during the current study are available from the corresponding author on reasonable request. They are uploaded as supplementary date in an excel sheet

Field-specific reporting

Please select the one below that is the best fit for your research. If you are not sure, read the appropriate sections before making your selection.

☒ Life sciences ☐ Behavioural & social sciences ☐ Ecological, evolutionary & environmental sciences

For a reference copy of the document with all sections, see nature.com/documents/nr-reporting-summary-flat.pdf

Life sciences study design

All studies must disclose on these points even when the disclosure is negative.

Sample size	40 patient samples
Data exclusions	no exlclusion data
Replication	repliable if posible three times when enough samples
Randomization	not relevant
Blinding	blinded by CHU Lille

Reporting for specific materials, systems and methods

We require information from authors about some types of materials, experimental systems and methods used in many studies. Here, indicate whether each material, system or method listed is relevant to your study. If you are not sure if a list item applies to your research, read the appropriate section before selecting a response.

Materials & experimental systems

n/a	Involved in the study	
☒	☐	Antibodies
☒	☐	Eukaryotic cell lines
☒	☐	Palaeontology and archaeology
☒	☐	Animals and other organisms
☒	☐	Human research participants
☐	☒	Clinical data
☒	☐	Dual use research of concern

Methods

n/a	Involved in the study	
☒	☐	ChIP-seq
☒	☐	Flow cytometry
☒	☐	MRI-based neuroimaging

Clinical data

Policy information about clinical studies

All manuscripts should comply with the ICMJE guidelines for publication of clinical research and a completed CONSORT checklist must be included with all submissions.

Clinical trial registration

ID: NCT04367142

Study protocol

This is a proof-of-concept biological diagnostic study carried out on the first nasopharyngeal swab collected from patients admitted to Lille University Hospital (emergency, hospitalization, resuscitation) for suspected COVID-19 infection, and also on saliva sputum collection at the time of nasopharyngeal sampling. Samples were collected systematically during the classical diagnostic management of COVID-19 by first PCR. PCR was performed according to the usual management. Sensor analysis was performed on the same first specimen as the PCR and on saliva collected by sputum. Any other PCR specimens that may be collected as part of the diagnostic procedure was not used to validate the sensor. The patient received the usual diagnostic and therapeutic management, apart from saliva collection. This analysis has therefore no impact on the individual as there was only one saliva collection per additional sputum and there will be no impact on diagnostic or therapeutic management.

This is a research mentioned in 3° of article L. 1121-1 of the public health code because there is no contact by essence with the in vitro diagnostic medical device, nor any change in the diagnostic or therapeutic conduct.
There is no risk or constraint associated with the realization of this biological proof of concept.
The samples are taken as part of the care, for the specific needs of the research there is only:
- An in vitro diagnosis of the samples by the CORDIAL-S in parallel with these samples.

Inclusion criteria:
- Male or female or child without age limit
- Admitted to a Reference Health Establishment (RHS) in an emergency unit, hospitalization or intensive care unit for suspicion of SARS-CoV-2 infection regardless of clinical presentation and degree of severity.
- Patient to be diagnosed by PCR test on nasopharyngeal swab.

	- Social insured<br><br>- A prospective collection of medical data (without additional constraints).<br>- A collection of saliva by sputum
Data collection	CHU Lille
Outcomes	90% positive and 90% negative percentage agreement on clinical samples, as compared to RT-PCR (i.e. representing the sensitivity and specificity since RT-PCR is the gold standard).
