# Supplementary material for: SARS-CoV-2 detection using a nanobody-functionalized voltammetric device
Source: Commun Med (Lond). 2022 May 23;2:56. doi: 10.1038/s43856-022-00113-8 (PMC9126950; doi:10.1038/s43856-022-00113-8)
Supplement: Supplementary file 4 — Description of Additional Supplementary Files [file 43856_2022_113_MOESM4_ESM.pdf]

Description of Additional Supplementary Files

File Name: Supplementary Data 1

Description: experimental data
